# Cyanophycinase is required for heterotrophy in cyanobacteria

**DOI:** 10.1016/j.jbc.2025.110791

**Published:** 2025-10-07

**Authors:** Éva Kiss, Martin Moos, Jan Mareš, Stanislav Opekar, Lenka Tomanová, Paulina Duhita Anindita, Martin Lukeš, Petra Urajová, Roman Sobotka

**Affiliations:** 1 Centre Algatech, Institute of Microbiology of the Czech Academy of Sciences, Třeboň, Czech Republic; 2 Institute of Entomology, Biology Centre of the Czech Academy of Sciences, České Budějovice, Czech Republic; 3 Faculty of Agriculture and Technology, Department of Applied Chemistry, University of South Bohemia, České Budějovice, Czech Republic; 4 Institute of Hydrobiology, Biology Centre of the Czech Academy of Sciences, České Budějovice, Czech Republic; 5 Faculty of Science, University of South Bohemia, České Budějovice, Czech Republic

**Keywords:** *Synechocystis*, cyanobacteria, heterotrophy, cyanophycinase, CphB, cyanophycin catabolism, arginine biosynthesis, ArgD, metabolic regulation, nitrogen/carbon homeostasis

## Abstract

Cyanophycin is a biopolymer of arginine (Arg) and aspartate, and it is found in various prokaryotes. Two key enzymes of cyanophycin metabolism are cyanophycin synthase (CphA), producing cyanophycin, and cyanophycinase (CphB), catalyzing the first step of cyanophycin degradation. CphB is a well-conserved enzyme found in the majority of cyanobacteria and ubiquitous amongst those that are known to perform heterotrophy besides their primary photosynthetic lifestyle. Unlike in diazotrophs, where CphB is connected to the mobilization of fixed nitrogen, the importance of this enzyme remains elusive in nondiazotrophs, such as the model cyanobacterium *Synechocystis* sp. PCC 6803. The *Synechocystis* Δ*cphB* deletion strain does not accumulate cyanophycin and shows no photoautotrophic growth defect. However, we show here that Δ*cphB* is not able to proliferate heterotrophically, although the CphA-less strain exhibits no obvious defect under heterotrophic conditions. Metabolomics profiling revealed that Δ*cphB* failed to upregulate the biosynthesis of Arg and displayed misregulated carbon and nucleoside metabolisms. These suggest that CphB is needed for the activation of the Arg pathway, which appeared to be crucial for balancing the nitrogen and carbon ratio during the acclimation to heterotrophy. On the other hand, the interaction of CphB with the Arg biosynthetic enzyme, acetylornithine aminotransferase, stimulated the hydrolysis of cyanophycin in an *in vitro* assay. These data, together with the metabolic profiles of Δ*cphB*, imply that the catabolism of cyanophycin and the biosynthesis of Arg are mutually coregulated metabolic pathways.

Cyanobacteria are highly adaptive microorganisms with remarkably flexible metabolism, which accounts for their vast abundance in versatile environments on Earth. For instance, many cyanobacteria are able to fix atmospheric N_2_, a trait that was dynamically gained and lost during their evolution (1). Moreover, they possess two distinct photosystems driving the extraction of electrons from water with the concomitant release of oxygen. This is one of the most demanding reactions in biology, and it allows cyanobacteria to produce vast amounts of ATP and NADPH to support the utilization of inorganic carbon (C) sources, such as atmospheric CO_2_. Being the only prokaryotes performing oxygenic photosynthesis, they have to manage especially diverse anabolic and catabolic processes in one compartment, which brings up the need for tight control over the different metabolic pathways. At the same time, many cyanobacteria are able to acclimate to facultative, nonphotosynthetic growth mode and proliferate solely on organic C sources (reviewed in Ref. (2)). The heterotrophic abilities of cyanobacteria recently gained attention in biotechnological applications (3) to optimize the production of targeted compounds (4, 5, 6). Despite the ecological and biotechnological importance of cyanobacteria, the regulation of their complex metabolism is poorly understood, even in the most widely studied *Synechocystis* sp. PCC 6803 (hereafter *Synechocystis*) (7). This cyanobacterium was originally isolated as a glucose-sensitive strain; however, several glucose-tolerant substrains have later become popular laboratory models (8).

*Synechocystis* is a nondiazotrophic, facultative photoautotroph (PAT), which, besides its principal, photosynthetic lifestyle, is able to grow heterotrophically utilizing organic molecules as energy, electron, and C source (2). For its dark, heterotrophic growth, *Synechocystis* needs a daily light pulse, which does not activate photosynthesis but has regulatory means (9). During this so-called light-activated heterotrophic (LAH) growth, *Synechocystis* efficiently assimilates glucose, and even though it grows substantially slower than photoautotrophically, the rate of protein synthesis remains comparable in LAH- compared with PAT-grown cells (10). On the other hand, the relative abundances of various proteins change intensively during the transition from PAT to LAH (10, 11) to acclimate the entire metabolism for heterotrophy. While in PAT, the major glycolytic pathways essentially serve as anaplerotic reactions, reinforcing C fixation (12); in LAH, the cells rely on external glucose, which is predominantly metabolized *via* the oxidative pentose phosphate (OPP) pathway (13, 14, 15, 16, 17). However, the light pulse–dependent activation of fructose-1,6-biphosphate (Fr-1.6-biP) aldolase, participating in the Embden–Meyerhof–Parnas (EMP) pathway, was also found to be crucial for the LAH glycolytic process (10, 18). Since the fundamental changes in C assimilation demand the adjustment of the intracellular C:nitrogen (N) ratio, enzymes involved in N metabolism also show significantly different cellular levels during LAH growth (11, 19). In particular, arginine (Arg) biosynthetic enzymes (see Fig. 1), such as acetylglutamate (AcGlu) kinase (ArgB), or acetylornithine aminotransferase (ArgD), are strongly upregulated in LAH conditions (11). Arg biosynthesis is a central target of the PII-regulatory pathways, signifying its importance in C/N homeostasis in cyanobacteria (reviewed in Ref. (20)). In *Synechocystis*, Arg is markedly channeled to the synthesis of a N-rich biopolymer (21), which forms granule peptide structures (cyanophycin granule peptide [CGP] (22, 23)). Similar to the Arg biosynthesis–related enzymes, cyanophycin synthase (CphA), which is responsible for the biosynthesis of CGP, also accumulates in LAH (11).

**Figure 1:**
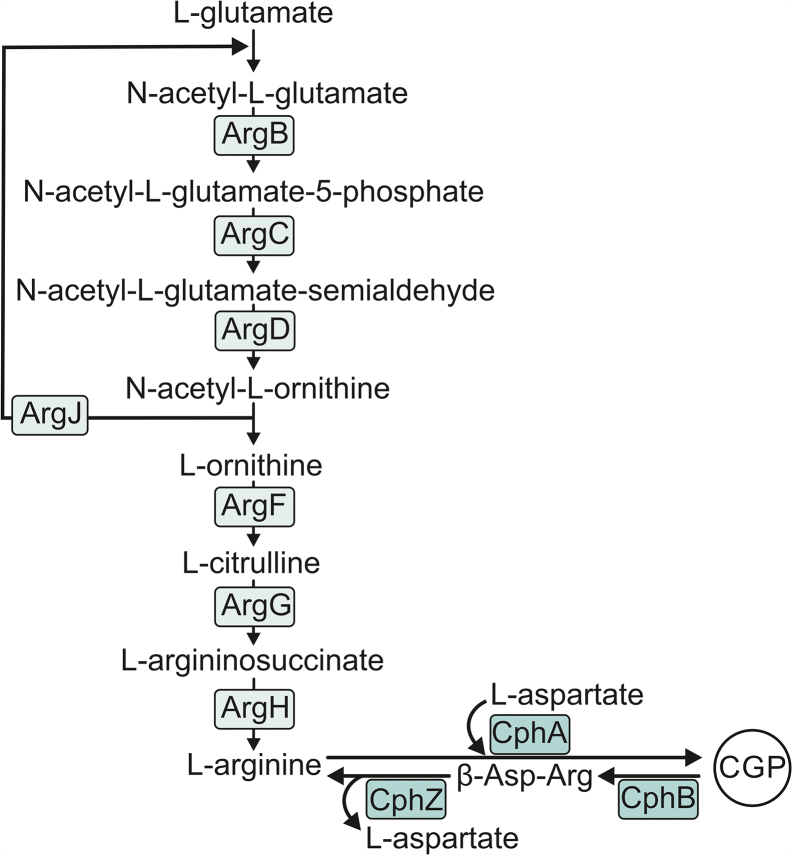
**Simplified scheme of the Arg and CGP biosynthetic pathways in cyanobacteria.** Glutamate is acetylated by glutamate/ornithine acetyltransferase (ArgJ), followed by phosphorylation catalyzed by acetylglutamate kinase (ArgB), and consequently, reduction to a semialdehyde form by *N*-acetyl-gamma-glutamyl-phosphate reductase (ArgC). The first four enzymatic steps, which are conserved from bacteria to plants (109), are accomplished by acetylornithine aminotransferase (ArgD). ArgJ then transfers the acetyl group from acetylornithine back to Glu. l-ornithine is converted into l-citrulline by ornithine carbamoyltransferase (ArgF). In the final steps of arginine biosynthesis, argininosuccinate synthase (ArgG) and lyase (ArgH) synthesize l-argininosuccinate and l-arginine, respectively. In cyanobacteria, substantial amounts of arginine can be channeled to the synthesis of cyanophycin granule peptide (CGP) (21) catalyzed by cyanophycin synthase (CphA). CGP is degraded to asparagine-arginine (β-Asp-Arg) dipeptide by cyanophycinase (CphB) and further hydrolyzed to l-arginine and l-aspartate by an isoaspartyl dipeptidase (CphZ). For more details, see Ref. (110).

CGP is catabolized to β-Asp-Arg dipeptides by cyanophycinase (CphB)—the CGP-specific exopeptidase (24). However, this enzymatic function appeared to be negligible, and the importance of CphB in *Synechocystis* remains to be elucidated (25). In the present study, we identified that CphB is needed at the early stages of acclimation to LAH, although cyanophycin metabolism had no direct role in this acclimation process. In fact, a CphB-dependent upregulation of Arg biosynthesis seems to be crucial at the onset of LAH for preventing the misregulation of the central C and nucleoside metabolisms. We further demonstrated that the interaction of ArgD with CphB (26) stimulates the *in vitro* hydrolysis of cyanophycin, suggesting a mutual coregulation of Arg biosynthesis and cyanophycin degradation.

## Results

### The CphB-null mutant of *Synechocystis* loses its viability under LAH

**Figure 2:**
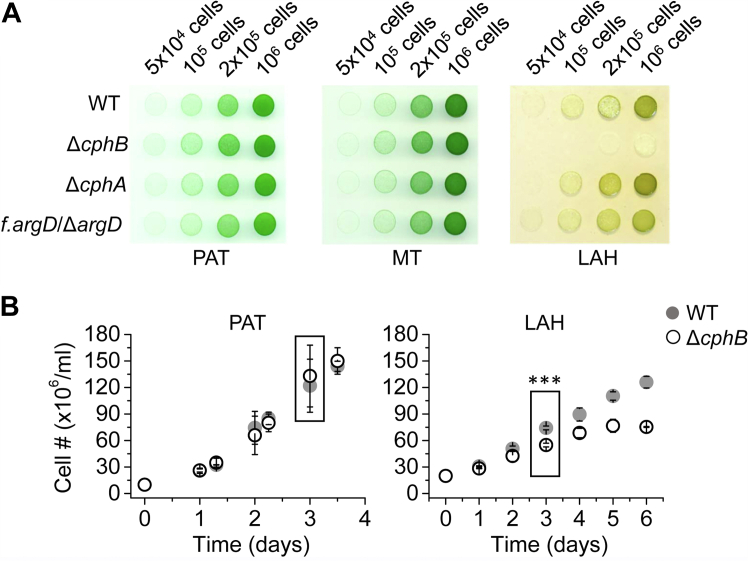
**CphB is required for heterotrophy.***A*, growth of the indicated strains in light with additional glucose (mixotrophic; MT), as well as under photoautotrophic (PAT), and light-activated heterotrophic (LAH) conditions. Batch cultures were exponentially grown under the control, PAT conditions. The concentration of cells in the cultures was adjusted, and the indicated number of cells was pipetted on solid media and cultivated further under PAT or transferred to MT or LAH conditions. *B*, changes in cell number in batch cultures of Δ*cphB* (*empty symbol*) and WT (*solid symbol*) grown under PAT or LAH. Symbols and error bars represent the average data of three independent experiments and their standard deviation, respectively. After 3 days of cultivation, the concentration of cells in the Δ*cphB* cultures was significantly lower (*p* < 0.001) compared with WT. This time point was chosen for sampling for further analysis (indicated by *squared boxes*). CphB, cyanophycinase.

To better understand the physiological function of CphB in cyanobacteria, we studied the effect of the elimination of this enzyme in the *Synechocystis* Δ*cphB* strain. As was shown earlier, the lack of CphB did not cause a growth defect in PAT ((27), Fig. 2). Since *Synechocystis* is able to proliferate on glucose in light (mixotrophic, MT) or in LAH growth conditions (2, 9), we tested whether Δ*cphB* shows phenotypic changes under these alternative, glucose-utilizing trophic modes. Remarkably, the Δ*cphB* cells hardly proliferate on agar plates with glucose in the dark (LAH), in contrast to their PAT and MT growth, which were comparable with the growth of the WT control strain (Fig. 2). The elimination of the CphA enzyme, which is responsible for the synthesis of CGP, had no effect on the LAH growth of the resulting strain (Δ*cphA*), implying that CGP is not crucial under these conditions (Fig. 2). The Δ*cphA* strain accumulated WT level of CphB (Fig. S1), further supporting that the absence of CphB is responsible for the LAH-growth defect. Monitoring WT and Δ*cphB* cells in liquid, batch cultures revealed that the proliferation of Δ*cphB* slowed down significantly (*p* < 0.001) from the third day of LAH cultivation; therefore, we used 3-day cultivated cell cultures for further analysis (Fig. 2B).

Cyanophycin and N metabolism seem to be interconnected, particularly in diazotrophic strains (25, 27). Besides, our data indicate that the CphB enzyme is needed for the heterotrophic growth mode in *Synechocystis* (Fig. 2). To test a potential relation between the presence of CphB and the competence in diazotrophy and/or heterotrophy, we analyzed the appearance of these traits in cyanobacteria. The capability to fix N_2_ was judged by the presence of the *nif* gene cluster (1). Since no particular gene(s) responsible for heterotrophy have been recognized so far (2), we narrowed down our analysis to species with available information about their trophic levels (Table S1).

**Figure 3:**
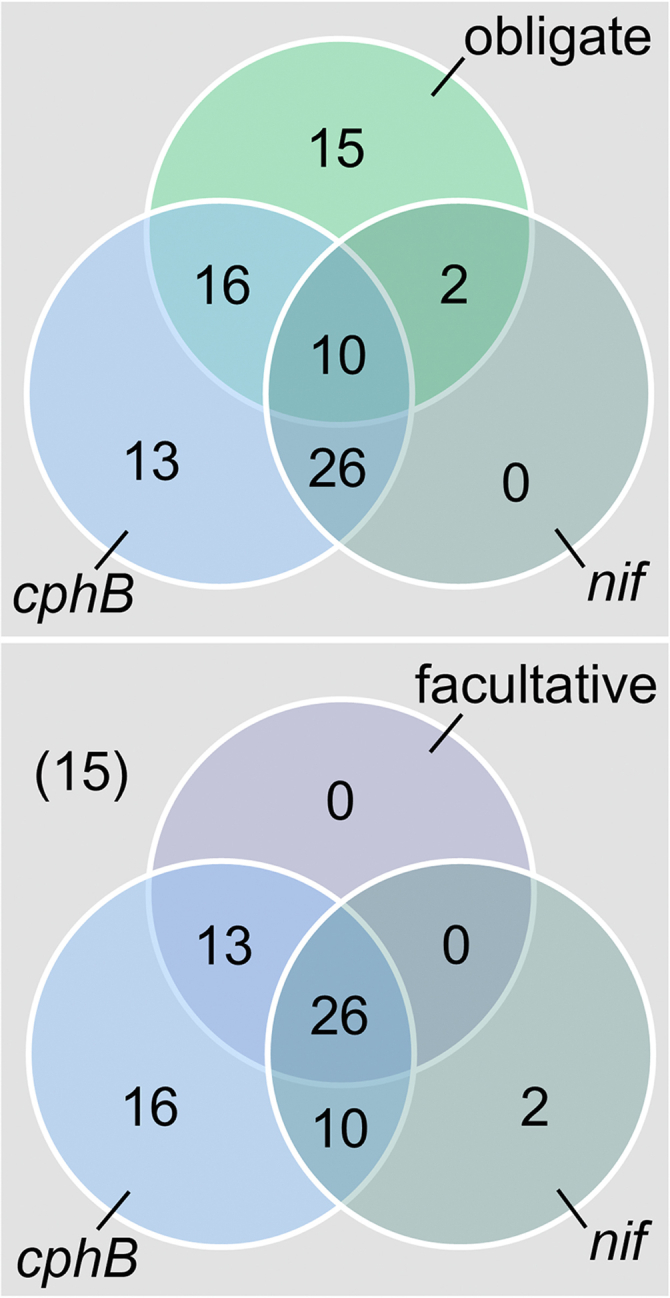
**Distribution ofthe cyanophycinase-encoding gene (*cphB*)and the nitrogenase-encoding gene cluster (*nif*) amongst obligate and facultative photoautotrophic cyanobacteria.** The Venn diagram was generated using a list of strains with known trophic levels (Table S1).

We found that the majority of cyanobacteria, even those in the most basal lineages, harbor CphB (Fig. S2). The evolution of the CphB protein in cyanobacteria resembles the evolution of conserved housekeeping proteins (28), further supporting that CphB is an ancestral enzyme. The CphB-encoding gene was lost only in five sublineages in our reconstructed phylogeny and was present in 65 of the 82 selected strains (Fig. S2). Even though cyanophycin metabolism seems to be primarily connected to N_2_ fixation (25, 27), CphB is widespread also amongst nondiazotrophic strains (29 strains contain *cphB* in the absence of *nif*; Fig. 3). Still, the majority of the CphB-containing species are able to fix N_2_ and/or to switch to nonphotosynthetic growth mode (*nif* and/or facultative: 49 of 65). Amongst those, many contain an additional open reading frame encoding a homolog of CphB (CphB-2, Fig. S2). The assumed gene duplication event leading to the origin of *cphB-2* was reconstructed to happen at roughly the same point in the evolution of cyanobacteria, at which the first strains capable of heterotrophic growth appeared. Notably, our analysis did not identify any strain capable of heterotrophy that would lack CphB (Fig. 3).

### Upregulation of Arg biosynthesis in LAH depends on CphB

**Figure 4:**
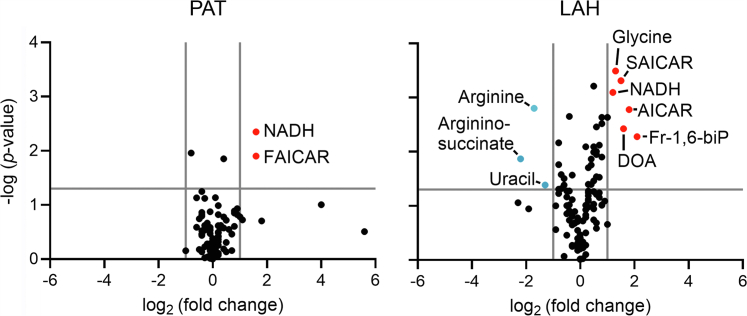
**Metabolic profiles of the Δ*cphB* and the WT control strains differ especially under LAH growth.** The selected metabolites used for the volcano plots were identified by LC–High Resolution (HR)MS and are listed in Table S2. Each data point was determined from the measurements of n = 3 samples derived from biologically independent experiments. The Welch's *t* test was used to test the null hypothesis. The significantly (*p* < 0.05) upregulated and downregulated (−1 > log_2_[fold change] >1) metabolites are indicated with labeled *red and blue symbols*, respectively. AICAR, 5-amino-4-imidazolecarboxamide; DOA, 2′- and 5′-deoxyadenosine; FAICAR, phosphoribosyl formamidocarboxamide; Fr-1,6-biP, fructose-1,6-biphosphate; PAT, photoautotrophic; LAH, light-activated heterotrophic; SAICAR, 1-(5′-phosphoribosyl)-5-amino-4-(succinocarboxamide)-imidazole.

The source of the heterotrophic growth defect of Δ*cphB* was analyzed by targeted metabolomics, focusing mainly on the central C and N pathways. The correlation between the *p* value and the fold change of the selected metabolites in PAT- and LAH-grown Δ*cphB* compared with WT is shown in volcano plots (Fig. 4). Under PAT conditions, two metabolites—NADH and 5-formamidoimidazole-carboxamide ribotide (a purine biosynthetic intermediate)—showed significantly higher accumulation in Δ*cphB* (Fig. 4). On the other hand, after 3 days of acclimation to LAH, when the mutant growth slowed down (Fig. 2), six metabolites showed significantly higher intracellular levels in Δ*cphB* compared with WT (Fig. 4). Moreover, Arg, and its biosynthetic intermediate, argininosuccinate, as well as uracil, were downregulated in the mutant (Fig. 4).

**Figure 5:**
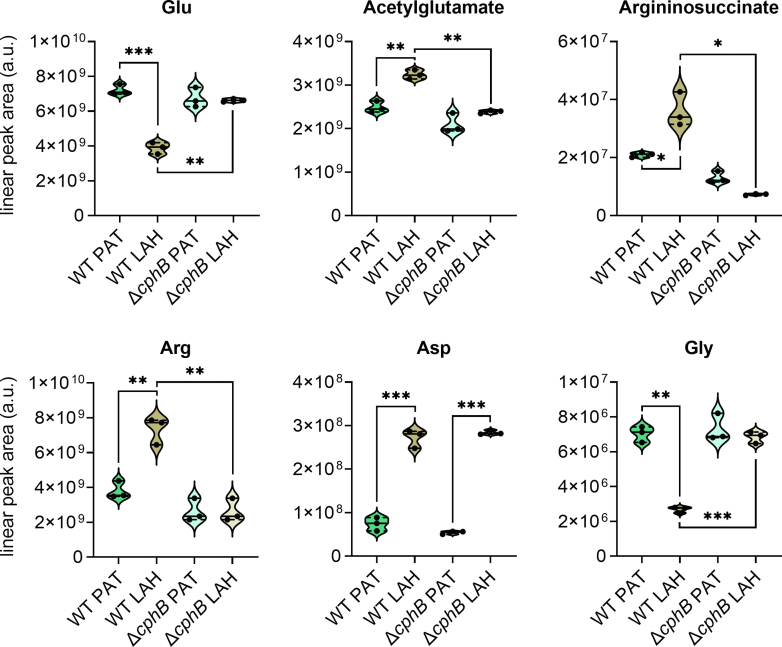
**Arg biosynthesis is upregulated in a CphB-dependent manner in LAH.** The relative amounts of glutamate, acetylglutamate, argininosuccinate, arginine (Arg), asparagine (Asp), and glycine (Gly) were determined by LC–High Resolution (HR)MS in samples prepared from equal amounts of WT control and cyanophycinase-less (Δ*cphB*) cells that were cultivated under photoautotrophic (PAT) or light-activated heterotrophic (LAH) conditions. The violin plots were generated from the data of three independent experiments, represented by *circles*. The median and the quartiles are indicated with *solid* and *dashed lines*, respectively. The Welch's *t* test was used to test the null hypothesis with a significance level set to *p* < 0.05. The significant differences are marked with ∗*p* < 0.05; ∗∗*p* < 0.01; ∗∗∗*p* < 0.001.

In bacteria, Glu is acetylated to AcGlu before entering the Arg biosynthetic pathway. We could see a dramatic drop in the relative amounts of Glu in LAH relative to PAT in WT but not in Δ*cphB* (Fig. 5). At the same time, the relative amount of AcGlu significantly increased in WT but not in Δ*cphB* (Fig. 5). These suggest that while in WT at least a partial amount of Glu is directed toward the synthesis of Arg; in Δ*cphB*, this initial step of Arg biosynthesis was less catalyzed. Δ*cphB* further showed a defect in the accumulation of argininosuccinate, a rate-limiting intermediate of Arg biosynthesis that was upregulated in WT after the transition from PAT to LAH (Fig. 5). Consequently, unlike in WT, the relative amount of Arg did not increase in the mutant in LAH (Fig. 5). Since the catalytic activity of CphB enables the release of Arg and Asp, the absence of this enzyme could potentially decrease Arg levels in the cells. However, Asp accumulated to a similarly high extent under LAH in both WT and Δ*cphB* (Fig. 5), further supporting that the relatively lower amount of Arg in the LAH-grown Δ*cphB* was due to a defect specifically in the biosynthesis of Arg.

Besides AcGlu kinase and ArgD, enzymes involved in the biosynthesis of aromatic amino acids were also upregulated in LAH (11). Our metabolomics data confirm the upregulation of tyrosine and tryptophan in both strains studied (Table S2). Apart from Arg, the most significantly changing amino acid was Gly, whose level was downregulated in LAH-grown WT (Fig. 5). Gly is primarily generated from the photorespiratory metabolite, 2-phosphoglycolate. Since photorespiration is expected to be negligible under nonphotosynthetic conditions, the significant drop in the Gly levels in LAH is comprehensible. However, in Δ*cphB*, the level of Gly in LAH did not follow the decrease observed in the WT but remained high, suggesting an impaired regulation of C metabolism in the mutant.

### Impaired utilization of carbohydrates in the absence of CphB

**Figure 6:**
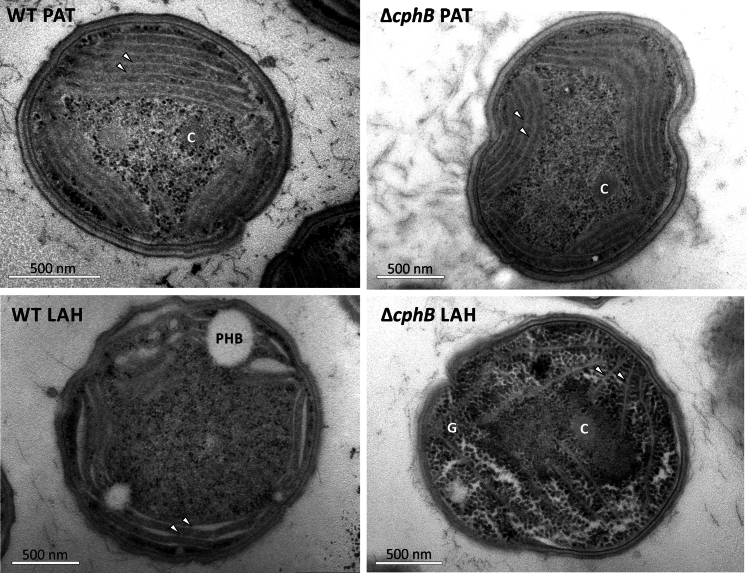
**Aberrant accumulation of carbon storage in the LAH-grown Δ*cphB* cells.** Electron micrographs were prepared of the WT and Δ*cphB* cells of *Synechocystis* grown for 3 days under PAT or LAH conditions. Similar images prepared from biologically independent cultures are shown in Fig. S3. The photosynthetic membrane lamellae are indicated by *arrows*; carboxysome (C), polyhydroxybutyrate (PHB), and glycogen (G) were identified according to the description of these inclusion bodies reviewed in Ref. (111). For images of excess glycogen accumulation in *Synechocystis,* see also Ref. (112). CphB, cyanophycinase; LAH, light-activated heterotrophic; PAT, photoautotroph.

It was previously reported that the deletion of CphB did not cause the accumulation of CGP in *Synechocystis* (25). We tested this conclusion under our experimental conditions by analyzing the cell ultrastructure using a transmission electron microscope before and after 3 days of acclimation to LAH. As reported before, the absence of CphB has no obvious effect in PAT conditions (25), and in LAH, the amount of photosynthetic membranes greatly reduces (19) (Figs. 6 and S3). Importantly, we could not observe CGP in any of the strains under the tested conditions, whereas the C storage material, glycogen, intensely accumulated in the LAH-grown Δ*cphB* (Figs. 6, S3 and S4).

Anoxic pathway of glucose breakdown was unlikely to be utilized, since the dissolved oxygen content in the LAH cultures remained relatively high (214 ± 8 μM). Also, the relative levels of one of the main products of fermentation, lactate, were presented in even lower levels in LAH compared with PAT conditions (Table S2). The early metabolites of glucose catabolism, such as the phosphate derivatives of glucose and fructose, although not significantly, consistently accumulated to a higher extent in the LAH-grown Δ*cphB* compared with WT (Fig. S4, Table S2). Both strains, but especially Δ*cphB*, contained relatively high amounts of 6- and 5-C sugar phosphates (Table S2, Fig. S4). On the other hand, the conversion of 6-C sugars to triose phosphates in the lower glycolytic path was apparently less efficient under LAH compared with PAT in both strains (Table S2, Fig. S4). Despite its slower growth under LAH (Fig. 2), the mutant had relatively higher levels of sugar phosphates, confirming that its LAH growth defect is not related to the availability of C for biomass production (Table S2, Fig. S4).

### Disrupted nucleotide homeostasis in the absence of CphB

**Figure 7:**
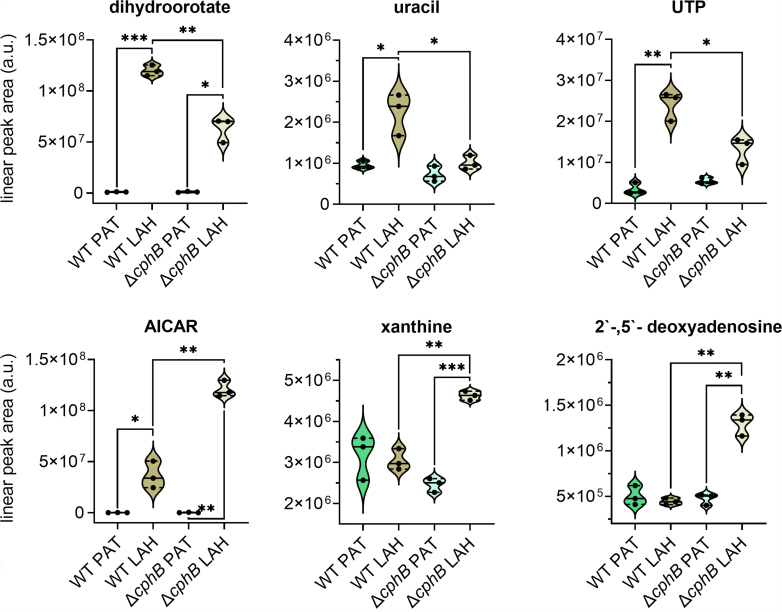
**Accumulation of nucleoside metabolites altered during the acclimation to LAH and in the absence of CphB.** For details, see Figure 5. AICAR, 5-amino-4-imidazolecarboxamide.

One of the most upregulated sugar phosphates in LAH was ribose-5-phosphate (R-5-P)—the early precursor of purine nucleotides. Its level increased in both strains but more dramatically in Δ*cphB* than in WT (Fig. S4). The high level of R-5-P was accompanied with higher levels of the biosynthetic intermediates of purines (such as 5-amino-4-imidazolecarboxamide, 1-(5′-phosphoribosyl)-5-amino-4-(succinocarboxamide)-imidazole, or 5-formamidoimidazole-carboxamide ribotide) in both strains grown under LAH conditions (Fig. 7, Table S2) but again, with a more significant increase in Δ*cphB* (Figs. 4 and 7, Table S2).

Unlike purines, dihydro-orotate and uracil, which are specific metabolites for the biosynthesis of pyrimidines, showed lower relative levels in Δ*cphB* (Figs. 4 and 7, Table S2). Consequently, the mutant contained less uridine triphosphate, the final product used for RNA synthesis (Fig. 7, Table S2). Moreover, although it accumulated more purine biosynthetic intermediates, the mutant failed to significantly upregulate the amount of the guanosine-triphosphate product (Table S2). The substantially lower ratios of nucleoside triphosphates/monophosphates in the LAH-grown mutant strongly suggest an impaired accumulation of nucleotides used for the biosynthesis of RNA (Fig. S5*A*). The unbalanced accumulation of purine and pyrimidine metabolites in Δ*cphB* was accompanied by a significant increase in the purine-degradation product, xanthine (Fig. 7). Notably, one of the most significantly upregulated metabolites in the LAH-grown Δ*cphB* was 2′- and 5′-deoxyadenosines (Figs. 4 and 7, Table S2).

### The ArgD enzyme enhances the activity of CphB *in vitro*

**Figure 8:**
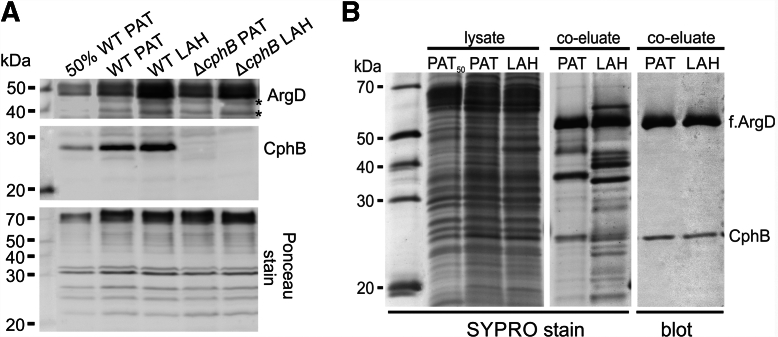
**ArgD binds CphB in both LAH and PAT conditions.***A*, the relative levels of ArgD and CphB under PAT and LAH in WT and Δ*cphB* were determined by immunodetection. Whole-cell lysates were separated on SDS-PAGE and blotted onto a PVDF membrane, and the ArgD and CphB proteins were detected using specific antibodies. The Ponceau staining of the membrane is shown for the loading control. Repetitions of the experiment and their statistical analysis are presented in Fig. S6. *B*, coimmunopurification of f.ArgD with CphB was performed using the same amounts of PAT- or LAH-grown *f.argD/*Δ*argD* cells. The eluates were separated by SDS-PAGE together with the input lysates, including 50% of the lysate from the PAT-grown cells (PAT_50_). The SYPRO-stained gel was subsequently blotted to a PVDF membrane, which was probed with specific antibodies against CphB and the FLAG tag of ArgD. The control f.ArgD pull-down prepared from the Δ*cphB* cells is shown in Ref. (26). CphB, cyanophycinase; f.ArgD, FLAG-tagged variant of ArgD; LAH, light-activated heterotrophic; PAT, photoautotroph; PVDF, polyvinylidene fluoride.

LAH-induced expression of Arg-biosynthetic enzymes was previously reported (11). We confirmed the increased level of ArgD in LAH, which was slightly, but not significantly (*p* = 0.165), less intense in Δ*cphB* (Figs. 8*A* and S6). As reported recently, the *Synechocystis* CphB binds to ArgD *in vivo* (26). To monitor the presence of ArgD–CphB complex during heterotrophy, we utilized a strain containing a FLAG-tagged variant of ArgD (f.ArgD) for pull-down assay; please note that the addition of an FLAG tag to ArgD had no observable effect under the tested conditions (Fig. 2). Although the accumulation of the f.ArgD–CphB complex can be induced by feeding with ornithine (28), f.ArgD copurified with CphB in a comparable ratio in PAT- and LAH-grown cells, suggesting that the formation of the complex is not affected by LAH conditions (Fig. 8*B*).

**Figure 9:**
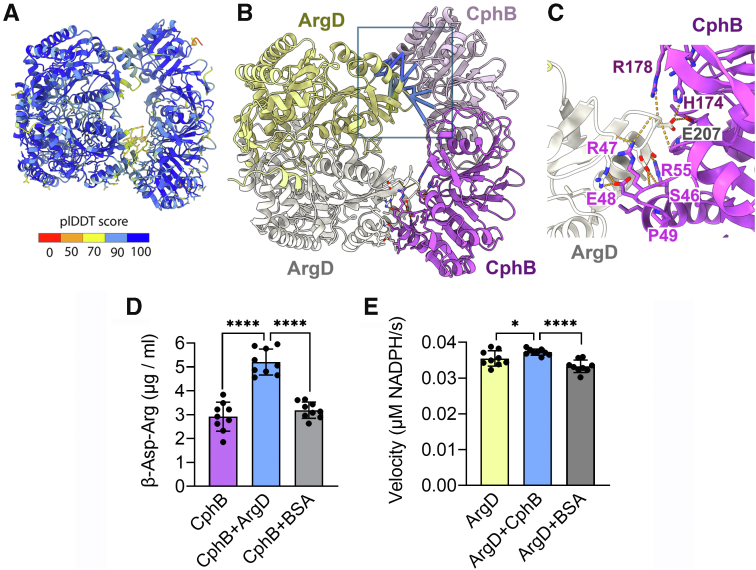
**ArgD binds CphB and stimulates its enzymatic activity in an *in vitro* assay.***A*, structural model of the ArgD_2_–CphB_2_ complex predicted by AlphaFold 3 (29) and colored according to the predicted local distance difference test (plDDT) score. An additional four models are shown in Fig. S7. *B*, representation of the individual polypeptides in the complex. The plDDT score for the contacts (up to a distance of 5 Å) between CphB and ArgD is shown within the *blue frame*. *C*, a detailed view of the interface between the CphB and ArgD proteins. The binding residues of CphB and ArgD are labeled in *magenta* and *gray*, respectively. The CphB residues participating in the β-(Asp-Arg)_2_ substrate binding are colored in *dark magenta* (30). Hydrogen bonds are highlighted as *yellow dashed lines*. All images were prepared in ChimeraX (61). *D*, *in vitro* activity of recombinant CphB (Fig. S9*A*) without or with ArgD in the reaction was assessed from its ability to degrade cyanophycin. The reaction was stopped at 120 s (Fig. S9*B*), and the generated β-Asp-Arg dipeptide was determined by ultra-high performance (UHP)LC–HRMS using a chemical standard. *E*, the *in vitro* activity of ArgD with or without CphB was determined in a coupled enzymatic reaction as described in Ref. (60). The generated NADPH fluorescence was measured and transformed into micromolar NADPH concentration using an NADPH standard curve (Fig. S9*C*). In *D* and *E*, the symbols, columns, and error bars represent the measurement of individual assays, their averaged data and standard deviation, respectively. For both assays, a control containing BSA instead of the respective interactive partner is shown. ∗∗∗∗*p* < 0.00001; ∗*p* = 0.017 as determined by two-tailed Student's *t* test. ArgD, acetylornithine aminotransferase; CphB, cyanophycinase.

In order to provide structural insight into the ArgD–CphB complex, we calculated an AlphaFold 3 prediction (29). Both ArgD and CphB are known to form homodimers (30, 31), so we focused on a hypothetical, tetrameric organization (ArgD_2_–CphB_2_). The calculated structure had very high confidence scores for almost all residues (predicted local distance difference test = 90–100), including the contact between ArgD and CphB (Fig. 9, *A*, *B* and S7). The Glu207 residue of ArgD forms hydrogen bonds with His174 and Arg178 of CphB (Fig. 9), which are also needed for the binding of the β-(Asp-Arg)_2_ substrate of CphB (30). We further checked how conserved the CphB (Ser46-Arg55) motif predicted to stabilize the CphB–ArgD assembly is. The analysis of CphB proteins from 1000 cyanobacterial strains revealed that those residues, which form a network of hydrogen bonds with ArgD (Ser46, Arg47, Glu48, and R55), are almost absolutely conserved (Fig. S8*A*). In contrast, the CphB enzymes from various bacterial strains lack such a motif (Fig. S8*B*).

Since the interaction of ArgD with the catalytic site of CphB is supported by high confidence in the AlphaFold 3 prediction, we assessed the activity of CphB in the absence or presence of ArgD, using recombinant proteins (Fig. S9*A*). Since the reaction was saturating in 4 min, we have chosen the 2 min time point for statistical analysis and quantified the concentration of the β-Asp-Arg dipeptide in the assay by LC–high-resolution mass spectrometry (HRMS) (Fig. S9*B*). Notably, the activity of CphB almost doubled if ArgD was added to the assay in a 1:1 M ratio (Fig. 9*D*). On the other hand, only 4% to 10% higher ArgD activity was measured when CphB was included in the reaction (Fig. 9*E*). These results indicate that the formation of the ArgD–CphB complex particularly enhances the catalytic rate of CphB.

## Discussion

### Regulation of Arg biosynthesis for heterotrophy

With CphA being amongst the most induced enzymes, cyanophycin metabolism is expected to be upregulated during LAH growth (11). Indeed, longer periods (2 w) of LAH cultivation on higher glucose supplements (60 mM) resulted in the accumulation of CGP (19). On the other hand, our data demonstrated that LAH growth was unaffected in Δ*cphA* (Fig. 2), which is unable to synthesize CGP (32) and has WT levels of CphB (Fig. S1). Moreover, we did not observe CGP accumulation in the LAH-cultivated Δ*cphB* mutant (Fig. 6). These imply that it is not the synthesis/turnover of CGP but the presence of CphB that is particularly crucial for heterotrophy.

The Arg biosynthetic enzymes show elevated expressions under LAH conditions (11), and the upregulated biosynthesis of Arg was pronounced in the LAH-grown WT, whereas it was absent in the Δ*cphB* mutant (Fig. 5). However, the *in vitro* activity of ArgD was only slightly affected by CphB (Fig. 9*E*), which can have very small, if any, biological significance. These signalize that if CphB controlled the Arg pathway *via* the complex with ArgD, additional factors are likely involved in the regulation. Alternatively, CphB targets different, yet unidentified, enzymatic step(s) in the pathway. As our attempts to tag CphB have failed, this scenario cannot be excluded. On the other hand, ArgD stimulated the CphB-catalyzed degradation of cyanophycin by twofold (Fig. 9*D*). This observation, together with the metabolic profile of the Δ*cphB* mutant, implies that the biosynthesis of Arg and the catabolism of cyanophycin are mutually coregulated. Since both these pathways lead to the accumulation of Arg, their coordination is compelling. While both CphB and ArgD are conserved enzymes amongst prokaryotes, the CphB segment predicted to bind ArgD is conserved in cyanobacteria but not in their bacterial counterparts (Fig. S8). These suggest that the interaction of ArgD with CphB, and potentially, the coregulation of CGP catabolism and Arg biosynthesis, is unique in cyanobacteria.

**Figure 10:**
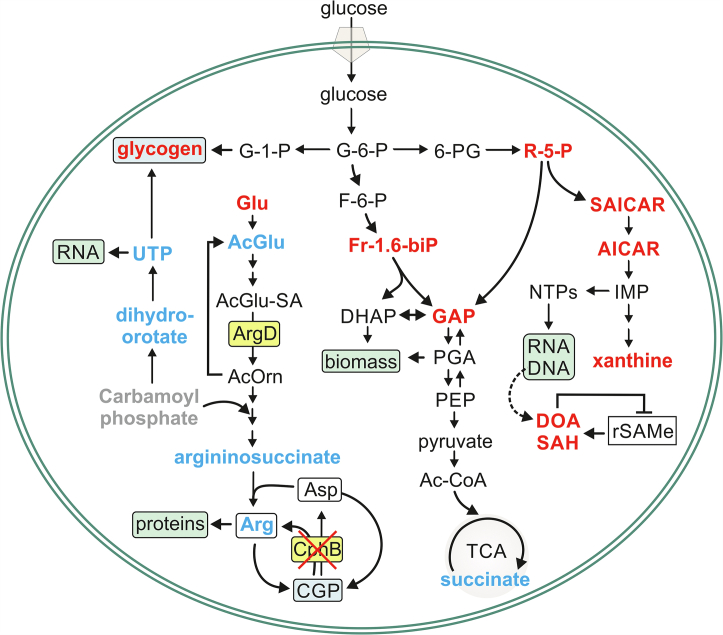
**A simplified scheme summarizing the LAH central metabolism of the Δ*cphB* cells compared with the WT control.** The metabolites indicated in *blue*, *red*, or *black* were showing significant decrease, increase, or no significant changes in Δ*cphB* compared with WT, respectively. The mutant lacking CphB channels much less Glu *via* AcGlu into Arg biosynthesis. We hypothesize that this regulatory defect causes further metabolic impairments, such as a hindered biosynthesis of pyrimidines and immense accumulation of the purine intermediates. The excess of purines is degraded as indicated by the accumulation of xanthine. Another prominent difference is in carbon metabolism, particularly the abnormal accumulation of the carbon storage material, glycogen, in the absence of CphB. In addition, the relatively high levels of toxic metabolites, such as 2′- and 5′-deoxyadenosine (DOA) and *S*-adenosyl-homocysteine (SAH), which inhibit the radical SAM enzymes (rSAMe), can have various detrimental effects on the metabolism of Δ*cphB*. See the main text for more abbreviations and the discussion for more details. DHAP, dihydroxyacetone phosphate; F-6-P, fructose-6-phosphate; G-1-P, glucose-1-phosphate; G-6-P, glucose-6-phosphate; LAH, light-activated heterotrophic; PEP, phosphoenolpyruvate; SA, semialdehyde.

Although the mechanism remains to be elucidated, the CphB-dependent upregulation of Arg biosynthesis was crucial for heterotrophy in *Synechocystis*. It has been previously recognized that Arg biosynthesis can be controlled at the level of various enzymes, such as ArgB, ArgD, and ArgG, signifying the importance of tight regulation over the pathway (33, 34, 35). Nevertheless, why *Synechocystis* needs to upregulate Arg metabolism during heterotrophy is unclear. A likely explanation after all stands for the previously suggested function of Arg metabolism in balancing C:N ratio, which has to be steadily controlled especially in phototrophs (discussed in Ref. (20)). The metabolism of LAH cells is entirely dependent on the organic C source in the growth medium (9), and although glucose is efficiently taken up by *Synechocystis*, it is mainly metabolized to C-5 rather than C-3 sugar phosphates ((15, 16), Table S2, Fig. S4). Consequently, relatively little glyceraldehyde-3-phosphate and 3-phosphoglyceric acid are available for biomass production in LAH (Fig. 10). Since *Synechocystis* maintains a high 5:1 ratio of C:N (20, 36), the limitation in C-3 sugar phosphates can potentially bring up the need to concomitantly decrease the amount of N available for protein synthesis. This can be efficiently carried out by the acetylation of Glu, which is then exclusively directed toward Arg biosynthesis.

### Metabolic deficiency of Δ*cphB* in heterotrophy

*Synechocystis* assimilates glucose in the form of glucose-6-phosphate (15), which accumulated to comparable levels in the WT and Δ*cphB* strains, indicating functional C uptake in both strains (Fig. S4). Glucose-6-phosphate is then predominantly metabolized *via* the EMP and OPP pathways that generate NADH and NADPH reductants, respectively (13, 14, 15, 16). The relatively higher amounts of NADH in the absence of CphB imply a more intense contribution of the EMP pathway to the breakdown of glucose (Table S2). A dominant role for the EMP pathway in Δ*cphB* is further suggested by the accumulation of Fr-1.6-biP, which is the most significantly upregulated sugar metabolite in the mutant (Fig. 4, Table S2, Fig. 10). These data further support that the activation of Fr-1.6-biP aldolase can represent a metabolic bottleneck during the acclimation to LAH (10, 18).

Concerning C metabolism, the most fundamental difference between the LAH-grown WT and Δ*cphB* cells was their glycogen content (Figs. 6, S3 and S4). It was previously reported that instead of CGP, glycogen was building up when CGP production was induced by chloramphenicol in a CphB-less *Synechocystis* strain (25). Similarly, we observed an aberrant accumulation of glycogen in the LAH-grown Δ*cphB* (Figs. 6, S3 and S4). Since the biosynthesis of Arg is coregulated with the mobilization of C storage (35), it is plausible that the impaired Arg biosynthesis in Δ*cphB* had a negative effect on the degradation of glycogen.

A likely explanation for the preference of OPP in LAH (13, 14, 15, 16) is to supply R-5-P, the sugar phosphate precursor for the purine and pyrimidine metabolites, which accumulate after the transfer to LAH. When the PAT cells face the altered trophic condition, they reshape their proteome to be able to rewire their entire metabolism to a different nutrient-acquisition mode (10, 11). Therefore, the acclimation process is expected to be strongly promoted by the transcriptional and translational machineries that are likely in need of a dynamic nucleotide metabolism, which is often key for the acclimation to growth challenges (37, 38). This could explain the excessive accumulation of metabolites in the pyrimidine, purine biosynthetic and salvage pathways, which showed some specific differences in Δ*cphB* (Fig. 7, Table S2). Most importantly, pyrimidines and their precursors (such as dihydro-orotate and uracil) were relatively less in Δ*cphB* (Fig. 7 and Table S2, Fig. 10). Moreover, uridine triphosphate, which is the final pyrimidine product specifically used for RNA synthesis, showed significantly lower levels. The pyrimidine and Arg biosynthetic pathways share their early carbamoyl phosphate precursors (Fig. 10), which synthesis has a high energy demand and is expected to be a crucial rate-limiting step of both pathways. We speculate that sharing a critical substrate brings up the need for coregulation of these biosynthetic routes, and a defect in the biosynthesis of Arg would affect the pyrimidine pathway. This is supported by the complex regulation of carbamoyl phosphate synthase by ornithine, uridine monophosphate, Arg, and pyrimidines (39, 40, 41).

Adversely to pyrimidines, the purine biosynthetic intermediates were relatively more abundant in the absence of CphB (Figs. 4 and 7, Table S2). However, despite the vast availability of the purine biosynthetic intermediates, the nucleotide triphosphate products for the synthesis of RNAs and DNA became limited in Δ*cphB*, especially when compared with the amount of their corresponding nucleoside monophosphate precursors (Fig. S5*A*). Consequently, the mutant failed to accomplish the nucleotide-demanding acclimation process and directed the excess amount of purine metabolites toward the degradation pathway (Figs. 7 and 10).

Deoxyadenosines were identified amongst the most significantly upregulated metabolites in the LAH-grown Δ*cphB* (Figs. 4 and 7). 2′- and 5′-Deoxyadenosines cannot be distinguished in our measurement, and the accumulation of both is plausible. 2′-Deoxyadenosine is a key purine metabolite and a critical component of DNA. The impaired nucleotide metabolism and proliferation of the mutant could potentially lead to DNA degradation and consequent accumulation of 2′-deoxyadenosine. While 5′-deoxyadenosine, along with *S*-adenosyl-homocysteine (Table S2), is a byproduct of the radical *S*-adenosyl methionine enzymes (reviewed in Ref. (42)). Nevertheless, deoxyadenosines, along with *S*-adenosyl-homocysteine, are metabolically toxic; their abnormal accumulation can obstruct fundamental cellular processes (42, 43). Overall, deletion of CphB led to severe metabolic deficiencies that likely increased oxidative stress (Fig. S5*B*) and eventually caused growth inhibition under LAH conditions.

All the strains capable of heterotrophy contain CphB, suggesting a general importance of this enzyme in the acclimation to an alternative, nonphotosynthetic growth mode (Fig. 3). However, our analysis was incomplete because of the limited information available about the trophic levels of cyanobacteria, which is related to the gap in our knowledge about the genes responsible for heterotrophy. Despite their evident importance, the metabolic processes driving the acclimation of a photosynthetic organism to heterotrophy are mainly unknown (2, 44). The present study signifies the complexity of the metabolic rearrangements that facultative cyanobacteria are capable of and urges more studies in this direction.

## Experimental procedures

### Construction and cultivation of *Synechocystis* strains

The glucose-tolerant *Synechocystis* substrain GT-P (45) was used as the WT control and as a genetic background for the preparation of mutants. The Δ*cphB*, *f.argD*/Δ*argD*, and the Δ*cphA* strains are described in Refs. (26) and (32), respectively. If not indicated otherwise, PAT cultures were grown in liquid BG-11 medium on a home-built rotary shaker at 28 °C, under continuous, moderate irradiance of 40 μmol photons m^−2^ s^−1^ given by white fluorescence tubes. For the transition to LAH, 3-day-old PAT cultures were washed to fresh BG-11 medium supplemented with 5 mM glucose to achieve a 4 × 10^7^ cells/ml density. The LAH cultures were agitated with 100 rpm on an orbital shaker (ELMI, catalog no.: S-3.02 20L) in darkness, combined with 5 min/24 h illumination with 40 μmol photons m^−2^ s^−1^. The dissolved oxygen content of the culturing media was determined by an immersible oxygen sensor (Presense, Fibox 4). The plate-drop experiments were performed by pipetting liquid cultures containing known concentrations of cells on BG-11 agar plates. The plates were color-scanned after cultivation under the indicated conditions. The stable pH of the solid media was ensured by 10 mM N-[Tris(hydroxymethyl)methyl]-2-aminoethanesulfonic acid. The number and average size of cells were assessed by Coulter counter (Multisizer 4; Beckman Coulter).

### Isolation and analysis of proteins and protein complexes

*Synechocystis* cells were mechanically broken as described in Ref. (26). Proteins in the whole-cell lysates were solubilized with β-dodecyl-n-maltoside. The insolubilized proteins were removed by centrifugation, whereas the solubilized proteins in the supernatant were loaded onto SDS-PAGE. The proteins were separated, visualized with SYPRO orange protein dye (Lumiprobe ProteOrange, catalog no.: 40210) and transferred onto a polyvinylidene fluoride membrane (Sigma–Aldrich, Immobilon-P, catalog no.: IPVH00010) that was subsequently incubated with primary anti-CphB and anti-ArgD antibodies (26). The primary antibodies were probed with anti-rabbit IgG–peroxidase antibody produced in goat (Sigma–Aldrich, catalog no.: A6154, Research Resource Identifier [RRID]: AB_11125345) and visualized using Immobilon Crescendo Western horseradish peroxidase substrate (Millipore, catalog no.: WBLUR0500, RRID: AB_439687) and luminescence image analyzer (Fuji, LAS-4000). The specificity of the primary antibodies was validated using strains containing the deletion and/or protein-tagged forms of the corresponding proteins (26). For assessing the apparent sizes of the proteins in the SDS-PAGE, a broad range, unstained protein ladder (Thermo Fisher Scientific, catalog no.: 26630) was used.

For the coimmunopurification assay, 2 l (10^8^ cells/ml) of *f.argD*/Δ*argD* cells (26) grown in PAT and LAH conditions were collected and mechanically broken. Soluble proteins were isolated by centrifugation and used for FLAG-affinity coimmunopurification assay (46). The protein coeluates were analyzed by immunodetection as described previously. f.ArgD was detected using anti-FLAG antibody (Sigma–Aldrich, catalog no.: F7425, RRID: AB_439687). The relative amounts of proteins in the lysates and coeluates were assessed from the intensity of the antibody signals using the ImageJ software (47). The band intensities were normalized to the corresponding loading control, such as SYPRO stain (Figs. 8*C*, S2 and S6) or Ponceau stain (Fig. 8), and the band intensity from the PAT-grown control WT was taken as one. The significance of the differences in the relative band intensities was assessed by a two-tailed Student's *t* test.

### Quantification of selected metabolites by LC–HRMS

Equal amounts of *Synechocystis* cells were pelleted from each examined culture and immediately frozen in liquid N_2_. Metabolites were extracted from the pellets by the addition of 100 μl of MeOH:acetonitrile (ACN):H_2_O (2:2:1 v/v/v) containing 4-fluorophenylalanine (1 nM/sample). The mixture was vortexed for 1 min, shock-frozen in liquid N_2_, and subsequently thawed in a thermoblock for 5 min at 30 °C. The sample was homogenized in an ultrasonic bath for 5 min at 0 °C, thoroughly mixed, and sonicated again under the same conditions. The mixture was centrifuged at 7000 rpm for 10 min at 4 °C, and the supernatant was collected. The homogenization (extraction) was repeated using 50 μl of MeOH:ACN:H_2_O (2:2:1 v/v/v). The gained supernatant was filtered through a 0.2 μm polyvinylidene fluoride minispin filter (HPST) at 8000 rpm for 10 min at 5 °C and was directly analyzed by LC–HRMS. An Orbitrap Q Exactive Plus mass spectrometer coupled to a Dionex Ultimate 3000 LC system (both from Thermo Fisher Scientific) was used for metabolite profiling, based on a previously published method (48). Chromatographic separation was performed using a SeQuant ZIC-pHILIC column (150 mm × 4.6 mm i.d., 5 μm; Merck KGaA) maintained at 35 °C. The mobile phase consisted of ACN and 20 mM aqueous ammonium carbonate with NH_4_OH to reach a pH of 9.2 (B). The flow rate was 450 μl/min, and the gradient program was as follows: 0 min, 20% B; 20 min, 80% B; 20.1 min, 95% B; 23.3 min, 95% B; 23.4 min, 20% B; and 30 min, 20% B. The injection volume was 5 μl. The mass spectrometer operated in full scan mode with a mass range of 70 to 1000 Da, a resolution of 70,000, and electrospray ionization in both positive and negative modes. Data were processed using Xcalibur software (version 4.0; Thermo Fisher Scientific) and an in-house developed Metabolite Mapper platform (see Table S3 for details).

The glycogen content of the cells was determined as described (49) with modifications established (50). Two milliliters of the samples were collected, spun down, and washed with distilled water. Cells were lysed by incubation in 400 μl of 30% KOH at 95 °C for 2 h. Glycogen was precipitated by the addition of 1 ml cold ethanol to a final concentration of 70% followed by an overnight incubation at −20 °C. The precipitated glycogen was pelleted by centrifugation at 15,000*g* for 10 min and washed with 70% ethanol and 98% absolute ethanol, consecutively. The precipitated glycogen was dried, then dissolved in 200 μl 100 mM sodium acetate (pH 4.5) containing 10 mg/ml (7 units) amyloglucosidase (10115; Sigma–Aldrich). The glycogen was digested at 60 °C for 2 h. The samples were subsequently mixed with 1 ml of 6% O-toluidine in acetic acid and incubated at 100 °C for 10 min. The absorbance was measured at 635 nm. A calibration curve prepared using different concentrations of glucose dissolved in sodium acetate was used to determine the amount of glycogen in the sample.

For the quantification of the selected metabolites, the average, median, and the standard deviation for each data point were determined from the measurements of n = 3 samples derived from biologically independent experiments. The Welch's *t* test was used to test the null hypothesis with a significance level set to *p* < 0.05. Statistical outliers were visually tested after plotting the dataset.

### Transmission electron microscopy

The ultrastructure of WT and Δ*cphB* cells grown for 3 days in PAT and LAH batch cultures was determined by transmission electron microscopy, which was performed as described in (51).

### Phylogenetic analysis

To assess the phylogenetic distribution of CphB, the *nif* gene cluster, and the ability to grow heterotrophically in cyanobacteria, a standardized phylogenomic species tree was constructed, utilizing the *de novo* workflow based on 120 concatenated conserved bacterial markers preselected in the Genome Taxonomy Database toolkit (52, 53). The Genome Taxonomy Database toolkit v2.3.0 release from May 2023 was used to produce a concatenated alignment (81 rows and 5035 amino acid positions in total) inferred from the set of cyanobacterial whole genomes of strains with known capability of photoautotrophic/heterotrophic growth (Table S1). The resulting alignment was utilized for phylogenetic inference using the maximum likelihood method under the GTR + I + G substitution model with 1000 ultrafast bootstrap replicates performed by IQTREE, v. 2.0.3 (54). The presence/absence of CphB and *nif* homologs in the target genomes was assessed using custom BLASTp searches against each genome using *slr2001* (CphB from *Synechocystis*) and several Nif proteins from *Nostoc* sp. PCC 7107 and *Pseudanabaena* sp. PCC 6802 as queries. The identity of the harvested BLAST hits was further verified by protein alignment in Geneious Prime 2020.0.3 software (www.geneious.com) and conserved domain analysis (55).

### Preparation of recombinant proteins

C-terminal His6-tagged CphB (*slr2001*; CphB-His), ArgD (*slr1022;* ArgD-His), and N-terminal Strep-tagged NADP-specific glutamate dehydrogenase (slr0710; STREPII-GdhA) proteins were overexpressed in *Escherichia coli* BL21 (DE3) using a pET21a plasmid (Fisher Scientific, catalog no.: 69-770-3) and the pET28a-RS plasmid derived from the pET28a according to Ref. (56), respectively. The expression was induced with 0.4 mM isopropyl-β-d-thiogalactopyranoside (Sigma–Aldrich, catalog no.: I5502) in exponentially grown cultures that were shaken for an additional 20 h at 18 °C. Cells were harvested by centrifugation (10 min, 4 °C, 10,000*g*) and resuspended in lysis buffer (20 mM Hepes [pH 8.0], 500 mM NaCl, 2 U/μl of benzonase nuclease [Millipore, catalog no.: 70664]) and protease inhibitor (SIGMAFAST Protease Inhibitor Cocktail Tablet, EDTA-Free, catalog no.: S8830). Cells were lysed mechanically on ice (seven cycles of 30 s sonication at 50% amplitude and 1 min off pulse). The lysate was clarified by centrifugation (4 °C, 1 h, 40,000*g*) and by filtration through a 0.22 μm PES membrane (Millipore, catalog no.: 99722). The clarified lysate containing CphB-His or ArgD-His was loaded into a Protino Ni–NTA 5 ml FPLC column (Macherey–Nagel Bioanalysis, catalog no.: 745415-5) using a Knauer FPLC system. Following sample loading, the column was washed with a buffer containing 20 mM Hepes, 500 mM NaCl, and 50 mM imidazole. CphB-His or ArgD-His was eluted from the column using elution buffer (20 mM Hepes, 500 mM NaCl, and 300 mM imidazole). For storage and further use, the eluted proteins were desalted using a HiTrap desalting 5 ml column (Cytiva, catalog no.: 17-1408-01) and stored in protein storage buffer (20 mM Hepes [pH 7.5], 150 mM NaCl, and 5% glycerol). For STREPII-GdhA purification, a Strep-Tactin XT 4Flow 1 ml FPLC column (IBA Lifesciences, catalog no.: 2-5023-001) was used, and the protein was eluted from the column using a buffer containing 20 mM Hepes (pH 8.0), 500 mM NaCl, 5% glycerol, and 50 mM biotin (IBA Lifesciences, catalog no.: 2-1016-005). Finally, proteins‘ purity was confirmed by SDS-PAGE on a 4% to 15% precast gel (Bio-Rad, catalog no.: 4561094) (Fig. S9*A*).

### Enzymatic assays

For the CphB activity assay, cyanophycin, purified from *Synechocystis* (57), was dissolved in 0.1 M HCl to reach a 1 mg/ml stock, which was used as a substrate for CphB in a 130 μg/ml final concentration. Degradation of cyanophycin by 2 μM CphB was carried out in the presence or absence of 2 μM ArgD, in 100 mM ammonium bicarbonate buffer (pH: 7.9) at 18 °C. The reaction was stopped by heat inactivation at 70 °C for 15 min (58). Thirty microliters of reaction mixture was diluted into 100 μl LC–MS grade water (Merck, catalog no.: 1.15333) and filtered on a 10 kDa cutoff spin column (Amicon Ultra; Millipore, catalog no.: UFC9010) at 4 °C, 14,000*g* for 45 min 50 μl of pass through was combined with 50 μl of LC–MS grade ACN (Merck, catalog no.: 1.00029) and directly analyzed *via* UHPLC (Agilent 1290 Infinity II UHPLC equipped with a diode array detector connected to HRMS with vacuum insulated probe heated electrospray ionization (timsTOF HT Mass Spectrometer; Bruker). The separation method used the following conditions: Acquity Premier BEH Amide column (2.1 × 150 mm, 1.7 μm) flow rate 0.4 ml/min, injection volume 1 μl, column temperature 45 °C, and mobile phase gradient: 0 min—95% A, 10.5 min—87% A, 12 min—60% A, 15 min—60% A, 15.1 min—95% A, and 20 min—95% A. The mobile phase A was ACN, and the mobile phase B was 15 mM aqueous ammonium acetate. Both phases contained 0.005% of acetic acid, as described (59). The mass spectrometry settings were as follows: dry temperature 230 °C; drying gas flow 8 l/min; sheath gas temperature 400 °C, sheath gas flow 4 l/min, nebulizer 2 bar; capillary voltage 4500 V; and endplate offset 500 V. The spectra were collected in the range of 20 to 1300 *m/z* with a 3 Hz rate. The collision energy was set to 20 eV. Calibration was performed using an internal calibration solution and CH_3_COONa clusters at the beginning of each analysis. The amount of the β-Asp-Arg product was assessed using a β-Asp-Arg dipeptide standard, which was synthesized by abcr GmbH. The activity of ArgD in the presence or absence of CphB was assessed from the formation of NADPH in a coupled enzymatic reaction as described (60). The generated NADPH was excited at 355 nm, and the emitted fluorescence was detected at 460 nm using a Fluostar Omega plate reader (BMG Labtech). The amount of NADPH formed during the coupled enzymatic reaction was extrapolated from an NADPH (Sigma–Aldrich, catalog no.: 53-57-6) standard curve. For both assays, the enzymes were preincubated for 30 min before the addition of the substrate, which was taken as time 0 in the activity measurements. The control reactions contained 1 mg/ml bovine serum albumin (Roth, catalog no.: 9048-46-8) instead of the corresponding interacting partner (ArgD or CphB). Data analyses were performed in GraphPad Prism 10 (GraphPad Software).

## Data availability

Metabolomics data have been deposited at figshare (https://doi.org/10.6084/m9.figshare.30067027.v1) and are publicly available as of the date of publication. Further information and requests for resources and reagents should be directed to and will be fulfilled by the corresponding author, Éva Kiss (kiss@alga.cz).

## Supporting information

This article contains supporting information with cited references (30, 47, 49, 50, 60, 61, 62, 63, 64, 65, 66, 67, 68, 69, 70, 71, 72, 73, 74, 75, 76, 77, 78, 79, 80, 81, 82, 83, 84, 85, 86, 87, 88, 89, 90, 91, 92, 93, 94, 95, 96, 97, 98, 99, 100, 101, 102, 103, 104, 105, 106, 107, 108).

## Conflict of interest

The authors declare that they have no conflicts of interest with the contents of this article.
