# Supplementary material for: Cyanophycinase is required for heterotrophy in cyanobacteria
Source: J Biol Chem. 2025 Oct 7;301(12):110791. doi: 10.1016/j.jbc.2025.110791 (PMC12661447; doi:10.1016/j.jbc.2025.110791)
Supplement: Supporting Tables and Figures [file mmc1.pdf]

SUPPORTING INFORMATION

Cyanophycinase is required for heterotrophy in cyanobacteria

Éva Kiss, Martin Moos, Jan Mareš, Stanislav Opekar, Lenka Tomanová, Paulina Duhita Anindita, Martin Lukeš, Petra Urajová, Roman Sobotka

This file includes Tables S1-3 and Figures S1 to S9.

Table S1 The list of cyanobacterial strains used for the assessment of the distribution of *cphB*, and the ability to fix N_2 and/or grow heterotrophically. *, absence (-) or presence (+) of the *nif* and *cphB* genes; **, obligate photoautotroph (can only utilize inorganic carbon sources); ***, facultative photoautotroph (capabale of utilizing inorganic as well as organic carbon sources).

**Organism**	***nif****	***cphB****	**trophic level**	**reference**
Acaryochloris marina MBIC11017	-	-	obligate**	(66,67)
Chamaesiphon minutus PCC 6605	-	+	obligate	(68,69)
Cyanobacterium aponinum PCC 10605	-	+	facultative***	(70,71)
Cyanobacterium stanieri PCC 7202	-	+	obligate	(69,72)
Cyanobium gracile PCC 6307	-	-	obligate	(68,69,72-76)
Cyanobium sp. PCC 7001	-	-	obligate	(69)
Gloeothece citriformis PCC 7424	+	+	obligate	(69) (77)
Crocosphaera subtropica ATCC 51142	+	+	facultative	(78,79)
Geminocystis herdmanii PCC 6308	-	+	obligate	(68,69,73)
Gloeobacter violaceus PCC 7421	-	+	obligate	(69)
Gloeocapsa sp. PCC 73106	-	+	obligate	(69)
Gloeocapsa sp. PCC 7428	-	+	facultative	(69)
Halothece sp. PCC 7418	+	-	obligate	(69,80)
Microcystis aeruginosa PCC 7806SL	-	+	obligate	(81)
Microcystis aeruginosa PCC 7005	-	+	obligate	(69,81)
Synechococcus elongatus PCC 6301	-	-	obligate	(68,69,82)
Synechococcus elongatus PCC 7942	-	-	obligate	(83,84)
Synechococcus elongatus PCC 6311	-	-	obligate	(69)
Synechococcus sp. PCC 6312	-	+	obligate	(68,69)
Parathermosynechococcus lividus PCC 6715	-	+	obligate	(69)
Picosynechococcus sp. PCC 73109	-	+	facultative	(69)
Picosynechococcus sp. PCC 7002	-	+	facultative	(69)

Synechococcus sp. PCC 7335	+	+	facultative	(69)
Synechococcus sp. PCC 7336	-	-	obligate	(85)
Synechococcus sp. PCC 7502	-	+	obligate	(69)
Synechocystis sp. PCC 6803	-	+	facultative	(68,69)
Synechocystis sp. PCC 7509	-	+	facultative	(69)
Chroococcidiopsis thermalis PCC 7203	+	+	facultative	(69,86)
Pleurocapsa sp. PCC 7319	-	+	facultative	(69,86)
Pleurocapsa sp. PCC 7327	+	+	facultative	(69,86)
Stanieria cyanosphaera PCC 7437	-	+	facultative	(69,86)
Xenococcus sp. PCC 7305	+	+	obligate	(69,86)
Coleofasciculus chthonoplastes PCC 7420	+	+	obligate	(69,87,88)
Baaleninema simplex PCC 7105	-	+	facultative	(69)
Geitlerinema sp. PCC 7407	-	+	facultative	(69)
Leptolyngbya boryana PCC 6306	+	+	facultative	(69,89,90)
Leptolyngbya sp. PCC 6406	+	-	obligate	(69,89,90)
Leptolyngbya sp. PCC 7375	+	+	obligate	(69)
Leptolyngbya sp. PCC 7376	-	+	facultative	(69)
Allocoleopsis franciscana PCC 7113	+	+	facultative	(69)
Nodosilinea nodulosa PCC 7104	+	+	facultative	(69,91,92)
Oscillatoria acuminata PCC 6304	-	+	obligate	(69)
Kaptonema formosum PCC 6407	+	+	facultative	(69)
Oscillatoria nigro-viridis PCC 7112	-	+	obligate	(69)
Kaptonema sp. PCC 6506	+	+	facultative	(69,89)
Pseudanabaena sp. PCC 6802	+	+	obligate	(68,69)
Pseudanabaena sp. PCC 7367	-	+	obligate	(69)
Pseudanabaena biceps PCC 7429	-	+	obligate	(69)
Spirulina major PCC 6313	-	+	obligate	(69)
Anabaena cylindrica PCC 7122	+	+	obligate	(69,93)
Anabaena sp. PCC 7108	+	+	obligate	(69,93)
Trichormus variabilis PCC 6309	+	+	obligate	(94)
Calothrix sp. PCC 6303	+	+	facultative	(69,73,94)
Calothrix sp. PCC 7103	+	+	facultative	(69,93)
Calothrix sp. PCC 7507	+	+	facultative	(69,93)
Cylindrospermum stagnale PCC 7417	+	+	facultative	(69,93)
Nostoc punctiforme PCC 73102	+	+	facultative	(69,93,95,96)
Nostoc sp. PCC 7107	+	+	facultative	(69,93)
Nostoc sp. PCC 7120	+	+	facultative	(69,93,97,98)
Nostoc sp. PCC 7524	+	+	facultative	(69)
Rivularia sp. PCC 7116	+	+	facultative	(69,93)
Prochlorococcus marinus str. MIT 9515	-	-	obligate	(99)
Prochlorococcus marinus str. MIT 9201	-	-	obligate	(100)
Chlorogloeopsis sp. PCC 7702	+	+	obligate	(31,69)
Chlorogloeopsis fritschii PCC 6912	+	+	facultative	(69)
Prochlorococcus marinus MIT9312	-	-	obligate	(101)
Prochlorococcus marinus MIT9211	-	-	obligate	(101)

Prochlorococcus marinus MIT9313	-	-	obligate	(101)
Prochlorococcus marinus MIT9303	-	-	obligate	(101)
Parasynechococcus marenigrum WH 8102	-	-	obligate	(102,103)
Synechococcus sp. CC9605	-	-	obligate	(104,105)
Trichodesmium erythraeum IMS101	+	+	obligate	(106,107)
Trichormus variabilis ATCC 29413	+	+	facultative	(69)
Synechocystis sp. PCC 6714	-	+	facultative	(69)
Fischerella muscicola PCC 7414	+	+	facultative	(69)
Fischerella muscicola PCC 73103	+	+	facultative	(69)
Fischerella thermalis PCC 7521	+	+	facultative	(69)
Fortiea contorta PCC 7126	+	+	facultative	(90)
Arthrospira platensis sp. PCC 7345	-	+	obligate	(69)
Thermosynechococcus vestilus BP-1	-	+	facultative	(108,109)
Scytonema hofmannii PCC 7110	+	+	facultative	(69)
Westiellopsis prolifica IICB1	+	+	facultative	(110)

Table S2 The relative fold changes of metabolites. The targeted metabolomics were carried out by LC-High Resolution (HR)MS in biological triplicates of WT and Δ*cphB* strains grown under photoautotrophic (PAT) and Light Activated Heterotrophic (LAH) conditions. The log_2 of the fold changes (log_2FC) induced by the absence of CphB under both conditions (LAH / PAT) and by the shift from PAT to LAH in both strains (ΔcphB / WT) are indicated. The statistical significance of the fold change, indicated by the *p*-value, was assessed by Welch's t-test.

**Metabolite Name**	**LAH, Δ*cphB* / WT**		**PAT, Δ*cphB* / WT**		**WT, LAH / PAT**		**Δ*cphB*, LAH / PAT**	
	**log_2FC**	***p*-value**	**log_2FC**	***p*-value**	**log_2FC**	***p*-value**	**log_2FC**	***p*-value**
L-Glutathione reduced	-2.3	0.0872	-0.2	0.9167	7.1	0.0538	5.0	0.0000
Argininosuccinic acid	-2.2	0.0135	-0.8	0.0110	0.7	0.0450	-0.7	0.0364
gamma-Glutamylcysteine	-1.9	0.1139	5.6	0.3106	15.1	0.0604	7.6	0.0033
L-Arginine	-1.7	0.0016	-0.6	0.0736	1.1	0.0047	0.0	0.5688
Uracil	-1.3	0.0411	-0.4	0.1609	1.4	0.0446	0.5	0.1316
Ribulose	-1.2	0.0170	-0.1	0.9265	-2.6	0.0011	-3.7	0.0005
Coenzyme A	-0.9	0.2754	0.3	0.2508	-5.8	0.0190	-7.0	0.0394
Orotic acid	-0.9	0.0995	-0.5	0.6612	3.8	0.0221	3.5	0.0028
L-Lysine	-0.8	0.0592	-0.4	0.3632	2.4	0.0221	2.0	0.0004
Isocitric acid	-0.8	0.0491	0.2	0.4754	2.3	0.0158	1.2	0.0004
Uridine 5'-triphosphate	-0.8	0.0174	0.9	0.1167	3.2	0.0044	1.5	0.0478
Dihydroorotic acid	-0.8	0.0068	-0.1	0.9695	6.8	0.0006	6.1	0.0122
Acetylphosphate	-0.7	0.0265	-0.3	0.9472	3.3	0.0011	2.8	0.0177
Succinic acid	-0.7	0.0480	0.1	0.4659	-2.2	0.0240	-3.0	0.0131
trans-Aconitic acid	-0.6	0.0312	0.2	0.7778	6.2	0.0049	5.4	0.0017
NADPH	-0.6	0.8544	4.0	0.0992	3.6	0.0676	-1.0	0.2885
Acetoacetic acid	-0.6	0.0411	0.8	0.1291	-0.7	0.1833	-2.1	0.0033
2-Oxoglutaric acid	-0.5	0.0532	0.8	0.1412	-0.9	0.1439	-2.2	0.0040
Oxaloacetic acid	-0.5	0.3468	-0.1	0.8454	2.9	0.0978	2.4	0.0000
Guanosine 5'-triphosphate	-0.5	0.1397	1.8	0.1969	4.2	0.0058	2.0	0.1321
Itaconic acid	-0.5	0.0684	0.0	0.5384	-0.6	0.0303	-1.1	0.0261
Cytidine triphosphate	-0.5	0.0989	1.1	0.1877	3.2	0.0175	1.7	0.0486
cis-Aconitic acid	-0.4	0.1237	0.1	0.6364	4.2	0.0129	3.7	0.0002
N-Acetyl-L-Glutamate	-0.4	0.0022	-0.3	0.0753	0.4	0.0019	0.3	0.1516
D-(+)-Glyceraldehyde	-0.4	0.0531	0.0	0.5412	0.0	0.8962	-0.4	0.1336
FAD	-0.4	0.1844	0.2	0.3177	0.1	0.8187	-0.5	0.1066
L-Tryptophan	-0.4	0.0961	-0.1	0.9017	0.8	0.0033	0.5	0.1955
FMN	-0.3	0.1372	0.3	0.2629	0.5	0.0709	-0.1	0.6019
(R)-Hydroxyglutaric acid	-0.3	0.0768	0.2	0.2406	4.1	0.0081	3.6	0.0018
L-Valine	-0.3	0.0270	-0.3	0.2397	2.2	0.0015	2.3	0.0003
L-Leucine	-0.3	0.1556	-0.3	0.2802	1.2	0.0148	1.2	0.0157
Phosphoenolpyruvic acid	-0.3	0.5613	0.8	0.2480	-2.9	0.0548	-4.0	0.0083
Uric acid	-0.3	0.5345	0.7	0.6942	-0.6	0.2217	-1.5	0.4288

5-Amino-2-oxopentanoate	-0.3	0.2243	-0.1	0.1335	-0.2	0.1412	-0.4	0.0541
L-Tyrosine	-0.2	0.1273	0.0	0.7070	1.2	0.0016	1.0	0.0012
Pyruvic acid	-0.2	0.2091	0.5	0.1918	-0.3	0.6182	-0.9	0.0640
Acetyl-CoA	-0.2	0.2044	0.0	0.9608	0.3	0.0497	0.1	0.8772
Carbamoyl acetate	-0.1	0.5393	-0.4	0.3490	-2.0	0.0684	-1.7	0.0589
L-Isoleucine	-0.1	0.4903	-0.4	0.2982	0.6	0.0423	0.9	0.0453
Fumaric acid	-0.1	0.2840	0.1	0.8014	-0.1	0.8785	-0.3	0.3432
L-Phenylalanine	-0.1	0.1936	-0.2	0.5245	-0.1	0.7709	0.0	0.5439
L-Alanine	-0.1	0.1667	0.0	0.9106	1.9	0.0003	1.7	0.0002
GSSG	-0.1	0.5147	0.1	0.0732	-2.0	0.0026	-2.2	0.0010
Malic acid	-0.1	0.4010	0.1	0.8001	-0.1	0.7963	-0.3	0.3243
L-Asparagine	-0.1	0.6623	-0.2	0.2814	-0.6	0.0004	-0.5	0.0152
NADP+	-0.1	0.5060	-0.1	0.6974	-1.1	0.0134	-1.2	0.0001
Glycerol-3P	-0.1	0.5699	0.2	0.1510	1.6	0.0064	1.4	0.0030
1-(5-Phospho-D-ribosyl)-5-amino-4-imidazolecarboxylate (CAIR) and 5-Carboxyamino-1-(5-phospho-D-ribosyl)imidazole	0.0	0.6242	0.6	0.2820	0.9	0.2368	0.3	0.8564
L-Homoserine	0.0	0.3415	0.0	0.5482	0.4	0.0144	0.4	0.1184
Propionyl-CoA	0.0		0.7	0.3458	-4.0	0.1133	-4.7	0.0878
L-Aspartic acid	0.0	0.4233	-0.4	0.1519	1.9	0.0003	2.3	0.0000
UDP-galactose	0.0	0.4422	0.1	0.4256	0.9	0.0015	0.8	0.0061
2-Hydroxydeoxy-ATP	0.0	0.3985	0.9	0.1508	1.1	0.0651	0.2	0.8470
L-Threonine	0.0	0.9759	-0.1	0.3375	0.8	0.0001	1.0	0.0045
ATP	0.0	0.3887	1.0	0.1647	1.1	0.0717	0.2	0.8215
L-Proline	0.0	0.6001	0.2	0.5556	-0.2	0.2243	-0.4	0.0446
Citric acid	0.0	0.5352	0.1	0.5018	0.6	0.0502	0.5	0.0127
CDP-glucose	0.0	0.9552	0.1	0.6690	0.4	0.1766	0.4	0.2906
L-Methionine	0.0	0.9595	-0.3	0.2907	-0.4	0.0896	-0.1	0.4503
Adenosine	0.1	0.1901	-0.1	0.5247	1.2	0.0017	1.4	0.0015
2'-Deoxyguanosine	0.1	0.1901	-0.1	0.5247	1.2	0.0017	1.4	0.0015
IMP	0.1	0.5959	0.2	0.3252	2.0	0.0087	1.9	0.0018
2'-Deoxyxanthosine 5'-monophosphate	0.1	0.5961	0.2	0.3252	2.0	0.0087	1.9	0.0018
AMP	0.1	0.4622	0.4	0.6502	-0.6	0.0611	-0.9	0.0204
Ribose	0.1	0.9407	0.0	0.6853	3.7	0.0119	3.9	0.0037
Ribulose-5-phosphate	0.2	0.1176	0.1	0.8051	0.7	0.0127	0.8	0.0041
Inosine	0.2	0.1477	-0.2	0.5476	4.7	0.0016	5.1	0.0014
5'-Deoxyxanthosine	0.2	0.1628	-0.2	0.5626	4.7	0.0017	5.1	0.0019
Hypoxanthine	0.2	0.0975	-0.4	0.3433	4.4	0.0016	5.0	0.0014
Phosphoribosyl formamidocarboxamide (FAICAR)	0.2	0.5293	1.6	0.0125	4.4	0.1015	3.0	0.0014
Nicotinic acid	0.2	0.3145	-0.2	0.2667	-1.2	0.0425	-0.8	0.0121
Uridine 5'-diphosphate	0.2	0.0957	0.4	0.1030	1.6	0.0012	1.4	0.0003

1-Methyl-3-pyrrolidinol	0.2	0.7930	-0.2	0.2163	-0.2	0.4049	0.3	0.5472
SAM	0.3	0.1050	0.1	0.5800	0.4	0.0080	0.6	0.0114
Glucose-1-phosphate	0.3	0.0570	0.0	0.4553	1.4	0.0096	1.6	0.0022
Adenine	0.3	0.0198	0.2	0.3862	1.3	0.0015	1.4	0.0003
Sedoheptulose-7-phosphate	0.3	0.0762	0.2	0.2422	2.4	0.0080	2.4	0.0028
Erythrose-4P	0.3	0.0648	0.0	0.6265	2.2	0.0187	2.5	0.0014
Glucose-6-phosphate	0.3	0.0801	0.0	0.5538	2.1	0.0234	2.4	0.0022
Ribose-5-phosphate	0.3	0.0400	0.0	0.5252	0.8	0.0029	1.1	0.0003
Glyceric acid	0.3	0.0529	0.4	0.0141	-1.5	0.0013	-1.6	0.0006
Phosphoric acid, Phosphate	0.4	0.0404	0.0	0.8111	-0.5	0.0169	-0.1	0.2834
Guanine	0.4	0.0176	-0.1	0.5984	0.2	0.5752	0.7	0.0095
Guanosine	0.4	0.0137	0.0	0.9929	0.2	0.5283	0.7	0.0085
2-(Formamido)-N1-(5'-phosphoribosyl)acetamidine (FGAM)	0.5	0.1133	0.2	0.8439	6.4	0.0229	6.6	0.0080
L-Lactic acid	0.5	0.0931	0.2	0.3765	-1.5	0.0177	-1.2	0.0010
SAH	0.5	0.0081	0.1	0.4425	-1.0	0.0420	-0.6	0.0013
NAD+	0.5	0.1818	0.3	0.2391	-0.4	0.2731	-0.2	0.1615
Thymine	0.5	0.0102	-0.4	0.0566	0.2	0.1114	1.1	0.0008
5-Methyltetrahydrofolate	0.5	0.0006	-1.0	0.7017	4.4	0.0005	5.9	0.0000
Sulfuric acid, Sulfate	0.5	0.0545	0.0	0.4163	-0.6	0.0306	-0.1	0.2998
D-Glyceraldehyde 3-phosphate	0.5	0.0346	0.0	0.8757	-0.9	0.0069	-0.4	0.0399
Guanosine diphosphate	0.6	0.0289	0.2	0.4311	0.7	0.0167	1.0	0.0116
Phosphoserine	0.6	0.0112	0.0		6.9	0.0059	7.5	0.0020
Xanthosine monophosphate	0.6	0.2058	0.0	0.9597	0.6	0.2435	1.1	0.0733
O-Phospho-L-threonine	0.6	0.2596	-0.5	0.1793	-0.3	0.3199	0.8	0.1490
Cytosine	0.6	0.0615	-0.6	0.2577	-1.9	0.0459	-0.8	0.0577
Sedoheptulose-1,7-diphosphate	0.6	0.0099	0.1	0.6742	1.1	0.0212	1.6	0.0005
Xanthine	0.6	0.0035	-0.4	0.1373	-0.2	0.7326	0.9	0.0002
Cytidine diphosphate	0.7	0.0571	0.1	0.6592	0.5	0.0828	1.1	0.0002
D-Glycerate-2-Phosphate	0.7	0.0697	0.5	0.4900	-2.7	0.0662	-2.5	0.0267
Guanosine 5'-monophosphate	0.7	0.0076	-0.3	0.5934	-0.9	0.0223	0.1	0.6322
L-Serine	0.7	0.0296	-0.1	0.2593	-2.2	0.0043	-1.4	0.0007
D-Glutamic acid	0.8	0.0030	-0.1	0.2920	-0.8	0.0002	0.0	0.7664
ADP	0.8	0.0973	0.4	0.1668	-0.8	0.0203	-0.5	0.0527
Uridine 5'-monophosphate	0.8	0.0081	0.2	0.2655	-0.6	0.0398	0.0	0.9160
Xanthosine	0.8	0.0023	0.5	0.7305	0.9	0.2279	1.3	0.0230
5'-Phosphoribosyl-N-formylglycinamide	0.8	0.0125	0.1	0.8313	2.3	0.0041	3.0	0.0050
6-phosphogluconic acid	0.9	0.0818	0.0	0.6558	1.2	0.0521	2.1	0.0280
Cytidine monophosphate	1.0	0.0023	-0.1	0.2874	1.0	0.0028	-0.1	0.4839
Aminoimidazole ribotide (AIR)	1.0	0.2189	0.1	0.4882	-0.3	0.1082	0.6	0.7115
NADH	1.2	0.0008	1.6	0.0045	-0.1	0.8623	-0.5	0.0525
Glycine	1.3	0.0003	-0.1	0.6390	-1.4	0.0013	0.0	0.4343

1-(5'-Phosphoribosyl)-5-amino-4-(N-succinocarboxamide)-imidazole (SAICAR)	1.5	0.0005	0.0		5.8	0.0181	7.3	0.0010
2'-Deoxyadenosine	1.6	0.0038	0.1	0.7060	-0.1	0.4438	1.4	0.0018
AICAR	1.8	0.0017	-0.4	0.7346	9.3	0.0413	11.5	0.0014
Fructose-1,6-diphosphate	2.1	0.0053	0.0	0.7258	0.0	0.6354	2.1	0.0041

Table S3 The list of metabolites detected by LC-HRMS in this study. Area 4500 were the instruments threshold of detection (electronic noise limit). If not indicated otherwise, the metabolites were identified by their match with MS/MS, the accurate mass, and the retention time (RT) of their specific chemical standards. NA, not available; PESI, NESI, positive or negative electrospray ionization, respectively. *, the metabolite was identified by its accurate mass match and spectrum match searched from the online metabolite library, METLYN (https://metlin.scripps.edu/landing_page.php?pgcontent=mainPage). **, accurate mass for $[M+H]^+$ or $[M-H]^-$ (< 3 ppm).

			HPLC/HRMS parameters		
**Metabolite name**	**Chemical formula**	**KEGG ID**	**RT [min]**	**Theoretic accurate mass****	**Ionization mode**
L-Glutathione reduced	C10H17N3O6S	C00051	10	308.0911	PESI
Argininosuccinic acid	C10H18N4O6	C03406	11.6	291.1299	PESI
gamma-Glutamylcysteine	C8H14N2O5S	C00669	9.3	251.0696	PESI
L-Arginine	C6H14N4O2	C00062	17.5	175.1190	PESI
Uracil	C4H4N2O2	C00106	5.4	111.0200	NESI
Ribulose	C5H10O5	C00309	7.3	149.0455	NESI
Coenzyme A*	C21H36N7O16P3S	C00010	9.0	766.1079	NESI
Orotic acid	C5H4N2O	C00295	7.0	107.0251	NESI
L-Lysine	C6H14N2O2	C00047	16.5	147.1128	PESI
Isocitric acid	C6H8O7	C00311	13.2	191.0197	NESI
Uridine 5'-triphosphate*	C9H15N2O15P3	C00075	12.6	482.9613	NESI
Dihydroorotic acid*	C5H6N2O4	C00337	7.3	157.0255	NESI
Acetylphosphate	C2H5O5P	C00227	10.0	138.9802	NESI
Succinic acid	C4H6O4	C00042	10.8	117.0193	NESI
trans-Aconitic acid	C6H6O6	C02341	13.6	173.0092	NESI
NADPH	C21H30N7O17P3	C00005	12.2	744.0838	NESI
Acetoacetic acid	C4H6O3	C00164	5.3	101.0244	NESI
2-Oxoglutaric acid	C5H6O5	C00026	11.0	145.0142	NESI
Oxaloacetic acid	C4H4O5	C00036	12.3	130.9986	NESI
Guanosine 5'-triphosphate	C10H16N5O14P3	C00044	11.3	521.9834	NESI
Itaconic acid	C5H6O4	C00490	10.7	129.0193	NESI
Cytidine triphosphate*	C9H16N3O14P3	C00063	12.6	481.9772	NESI
cis-Aconitic acid	C6H6O6	C00417	12.6	173.0092	NESI
N-Acetyl-L-Glutamate	C7H11NO5	C00624	10.0	188.0564	NESI
D-(+)-Glyceraldehyde*	C3H6O3	C00577	11.1	89.0244	NESI
FAD	C27H33N9O15P2	C00016	7.7	784.1499	NESI
L-Tryptophan	C11H12N2O2	C00078	7.8	205.0972	PESI
FMN*	C17H21N4O9P	C00061	7.2	455.0973	NESI
(R)-Hydroxyglutaric acid	C5H8O5	C01087	10.8	147.0299	NESI
L-Valine	C5H11NO2	C00183	8.4	116.0717	NESI

L-Leucine	C6H13NO2	C00123	6.8	132.1019	PESI
Phosphoenolpyruvic acid	C3H5O6P	C00074	12.5	166.9751	NESI
Uric acid	C5H4N4O3	C00366	8.4	167.0211	NESI
5-Amino-2-oxopentanoic acid*	C5H9NO3	C01110	9.6	132.0655	PESI
L-Tyrosine	C9H11NO3	C00082	8.4	180.0666	NESI
Pyruvic acid	C3H4O3	C00022	5.5	87.0088	NESI
Acetyl-CoA	C23H38N7O17P3S	C00024	8.4	810.1330	PESI
Carbamoyl_acetate*	C3H5NO3	NA	7.4	102.0197	NESI
L-Isoleucine	C6H13NO2	C00407	7.2	132.1019	PESI
Fumaric acid	C4H4O4	C00122	11.4	115.0037	NESI
L-Phenylalanine	C9H11NO2	C00079	6.3	166.0863	PESI
L-Alanine	C3H7NO2	C00041	9.9	90.0550	PESI
L-Glutathione oxidized, GSSG	C20H32N6O12S2	C00127	12.1	613.1592	PESI
Malic acid	C4H6O5	C00149	11.3	133.0142	NESI
L-Asparagine	C4H8N2O3	C00152	10.2	131.0462	NESI
NADP+	C21H29N7O17P3	C00006	11.2	742.0677	NESI
Glycerol-3P	C3H9O6P	C00093	10.4	171.0064	NESI
1-(5-Phospho-D-ribosyl)-5-amino-4-imidazolecarboxylate (CAIR) and 5-Carboxyamino-1-(5-phospho-D-ribosyl)imidazole*	C9H14N3O9P	C04751	12.6	338.0395	NESI
L-Homoserine	C4H9NO3	C00263	10.4	182.0812	PESI
Propionyl-CoA*	C24H40N7O17P3S	C00100	7.5	824.1487	PESI
L-Aspartic acid	C4H7NO4	C00049	10.4	132.0302	NESI
Uridine diphosphate galactose*	C15H24N2O17P2	C00052	11.6	565.0477	NESI
2-Hydroxydeoxy-ATP*	C10H16N5O13P3	C19969	11.2	505.9885	NESI
L-Threonine	C4H9NO3	C00188	9.9	120.0655	PESI
ATP	C10H16N5O13P3	C00002	11.9	505.9885	NESI
L-Proline	C5H9NO2	C00148	8.5	114.0561	NESI
Citric acid	C6H8O7	C00158	12.6	191.0197	NESI
CDP-glucose*	C15H25N3O16P2	C00501	15.0	564.0637	NESI
L-Methionine	C5H11NO2S	C00073	7.4	150.0583	PESI
Adenosine	C10H13N5O4	C00212	5.7	266.0895	NESI
2'-Deoxyguanosine*	C10H12N4O5	C00330	6.8	267.0735	NESI
Inosine 5'-monophosphate (IMP)	C10H13N4O8P	C00130	10.8	347.0398	NESI
2'-Deoxyxanthosine 5'-monophosphate*	C10H14N5O7P	NA	7.3	346.0558	NESI
AMP	C10H14N5O7P	C00020	9.7	346.0558	NESI
Ribose	C5H10O5	C00121	8.0	149.0455	NESI
Ribulose-5-phosphate	C5H11O8P	C00199	11.2	229.0119	NESI
Inosine	C10H12N4O5	C00294	7.0	267.0735	NESI
5'-Deoxyxanthosine*	C10H12N4O5	NA	6.8	267.0735	NESI
Hypoxanthine	C5H4N4O	C00262	6.8	135.0312	NESI
Phosphoribosyl formamidocarboxamide (FAICAR)*	C10H15N4O9P	C04734	6.8	365.0504	NESI
Nicotinic acid	C6H5NO2	C00253	4.8	124.0393	PESI

Uridine 5'-diphosphate	C9H13N2O9P	C00015	10.6	323.0286	NESI
1-Methyl-3-pyrrolidinol*	C5H11NO	NA	3.4	102.0913	PESI
S-Adenosylmethionine (SAM)	C15H22N6O5S	C00019	10.7	399.1445	PESI
Glucose-1-phosphate	C6H13O9P	C00103	11.4	259.0224	NESI
Adenine	C5H5N5	C00147	6.0	134.0472	NESI
Sedoheptulose-7-phosphate	C7H15O10P	C05382	11.4	289.0330	NESI
Erythrose-4P	C4H9O7P	C00279	11.6	199.0013	NESI
Glucose-6-phosphate	C6H13O9P	C00668	12.1	259.0224	NESI
Ribose-5-phosphate	C5H11O8P	C00117	11.3	229.0119	NESI
Glyceric acid	C3H6O4	C00258	7.8	105.0193	NESI
Phosphoric acid, Phosphate	H3PO4	C00009	11.3	96.9696	NESI
Guanine	C5H5N5O	C00242	8.3	150.0421	NESI
Guanosine	C10H13N5O5	C00387	8.4	282.0844	NESI
2-(Formamido)-N1-(5'-phosphoribosyl)acetamidine (FGAM)*	C8H16N3O8P	C04640	9.9	312.0602	NESI
L-Lactic acid	C3H6O3	C00186	6.3	89.0244	NESI
S-Adenosylhomocysteine (SAH)	C14H20N6O5S	C00021	8.8	385.1289	PESI
NAD+	C21H27N7O14P2	C00003	9.4	664.1164	PESI
Thymine	C5H6N2O2	C00178	5.8	125.0357	NESI
5-Methyltetrahydrofolate*	C20H25N7O6	C00440	9.6	460.1939	PESI
Sulfuric acid, Sulfate	H2SO4	C00059	12.5	96.9601	NESI
D-Glyceraldehyde 3-phosphate	C3H7O6P	C00118	11.2	168.9907	NESI
Guanosine diphosphate	C10H15N5O11P2	C00035	12.8	442.0171	NESI
Phosphoserine	C3H8NO6P	C01005	11.5	184.0016	NESI
Xanthosine monophosphate*	C10H13N4O9P	C00655	12.4	363.0347	NESI
O-Phospho-L-threonine	C4H10NO6P	C12147	11.4	200.0318	PESI
Cytosine	C4H5N3O	C00380	7.5	110.0360	NESI
Sedoheptulose-1,7-diphosphate*	C7H16O13P2	C00447	10.8	368.9993	NESI
Xanthine	C5H4N4O2	C00385	8.0	151.0261	NESI
Cytidine diphosphate*	C9H15N3O11P2	C00112	12.0	402.0109	NESI
D-Glycerate-2-Phosphate*	C3H7O7P	C00631	11.9	184.9857	NESI
Guanosine 5'-monophosphate*	C10H14N5O8P	C00144	11.8	362.0507	NESI
L-Serine	C3H7NO3	C00065	10.6	106.0499	PESI
D-Glutamic acid	C5H8O4	C00217	10.4	131.0350	NESI
ADP	C10H15N5O10P2	C00008	11.0	426.0221	NESI
Uridine 5'-monophosphate	C9H14N2O12P2	C00105	11.5	402.9949	NESI
Xanthosine	C10H12N4O6	C01762	8.5	283.0684	NESI
5'-Phosphoribosyl-N-formylglycinamide*	C8H16N3O8P	C04376	6.2	312.0602	NESI
6-phosphogluconic acid	C6H13O10P	C00345	12.5	275.0174	NESI
Cytidine monophosphate	C9H14N3O8P	C05822	11.1	322.0446	NESI
Aminoimidazole ribotide (AIR)*	C8H14N3O7P	C03373	9.7	294.0497	NESI
NADH	C21H29N7O14P2	C00004	8.8	664.1175	NESI
Glycine	C2H5NO2	C00037	10.6	76.0393	PESI

1-(5'-Phosphoribosyl)-5-amino-4-(N-succinocarboxamide)-imidazole (SAICAR)*	C13H19N4O12P	C04823	13.8	453.0664	NESI
2'-Deoxyadenosine	C10H13N5O3	C00559	4.7	250.0946	NESI
AICAR*	C9H14N4O8P	C04677	10.6	336.0476	NESI
Fructose-1,6-diphosphate	C6H14O12P2	C00354	13.2	338.9888	NESI

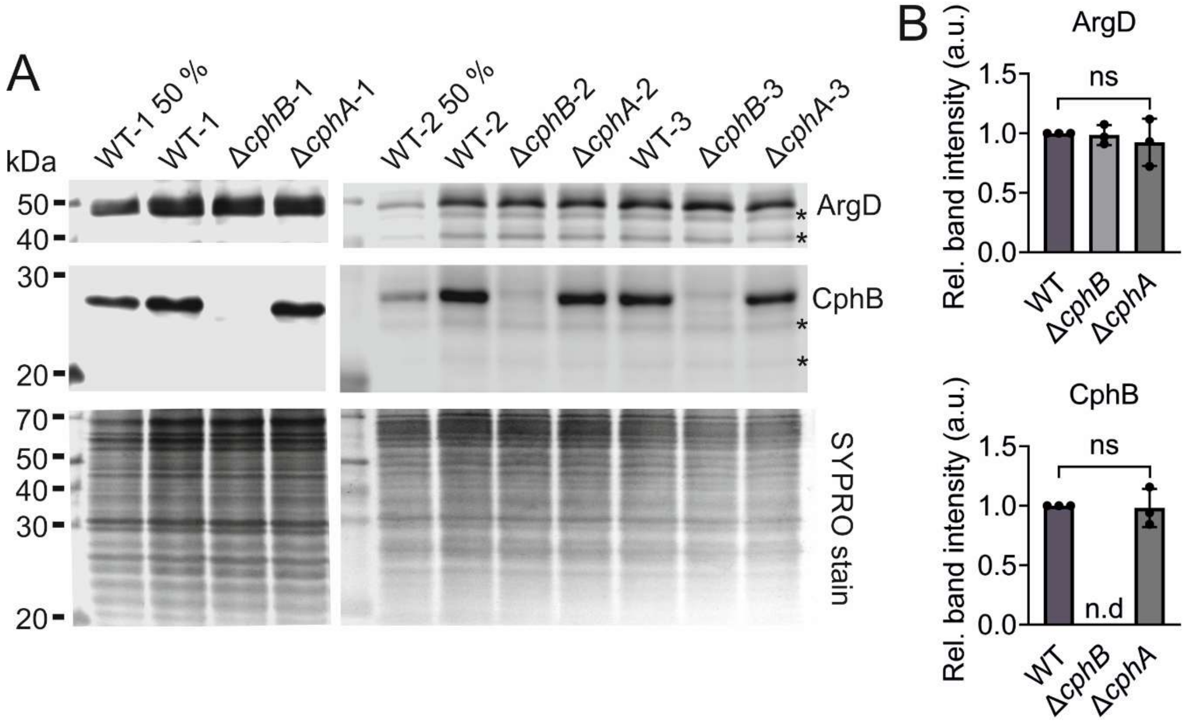

Figure S1 The deletion of cyanophycinase synthase (CphA; Δ*cphA*) does not interfere with the intracellular accumulation of cyanophycinase (CphB). *A*, Lysates prepared from equal amounts of WT, Δ*cphB*, and Δ*cphA* cells were analysed by protein immunoblot using specific antibodies against the ArgD and CphB enzymes. Preparation from biologically independent cultures (designated 1 to 3) are shown. The corresponding part of the stained gel (SYPRO stain) was used for loading control. *, unspecific cross-reaction *B*, Relative band intensities of the ArgD and CphB proteins presented on panel *A*. Each band intensity was normalized to its corresponding loading control. The symbols, columns and error bars represent the relative values generated from biologically independent cultures, their average and standard deviation, respectively. The significance of the differences was tested with 2-tailed Student's *t*-test. ns, not significant; nd, not detected.

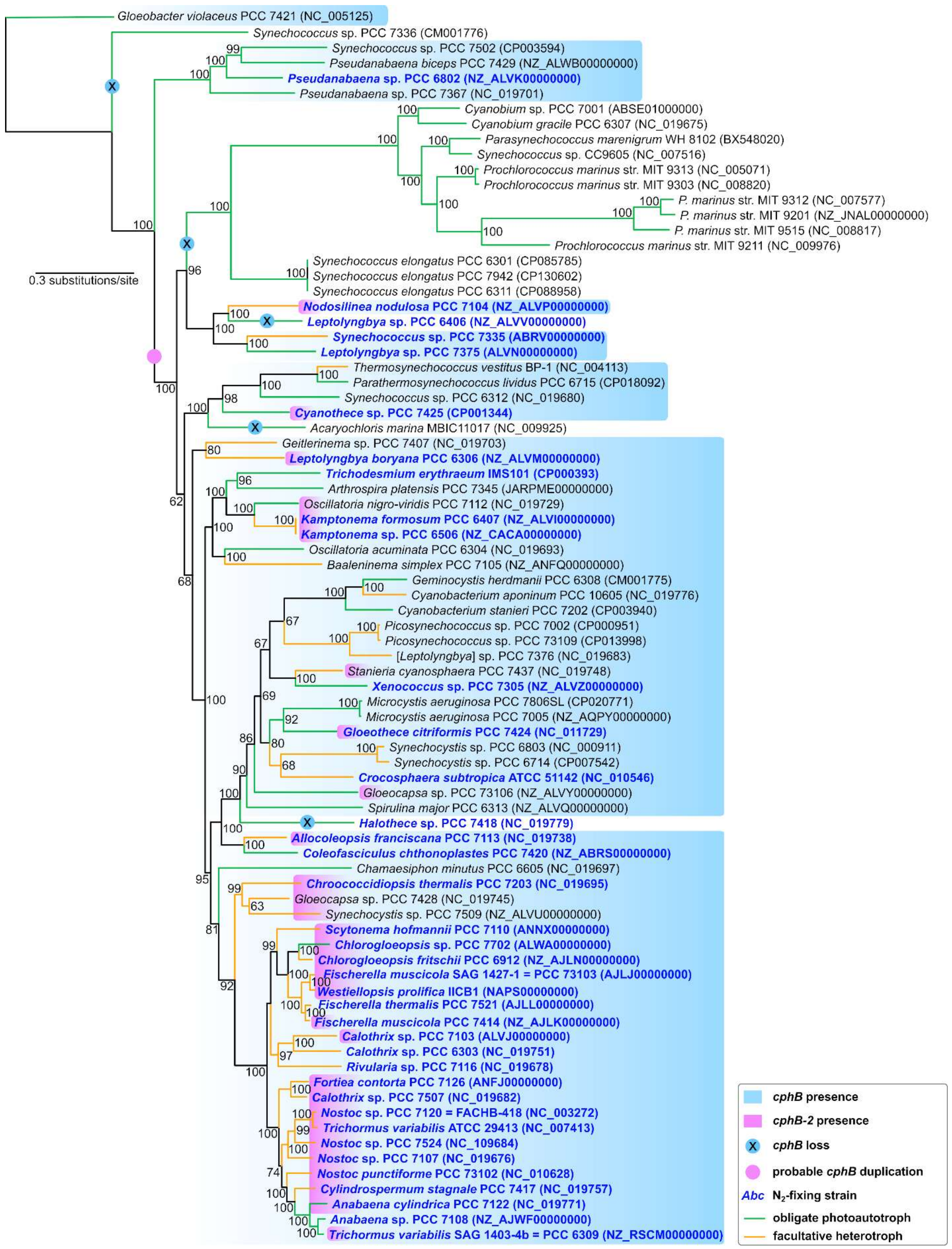

Figure S2 Phylogenomic species tree mapping the evolutionary correlation among the presence of the *cphB* and *cphB-2* homologues, N_2-fixation, and facultative photoautotrophy in cyanobacteria. The bootstrap values (≥ 50) are indicated.

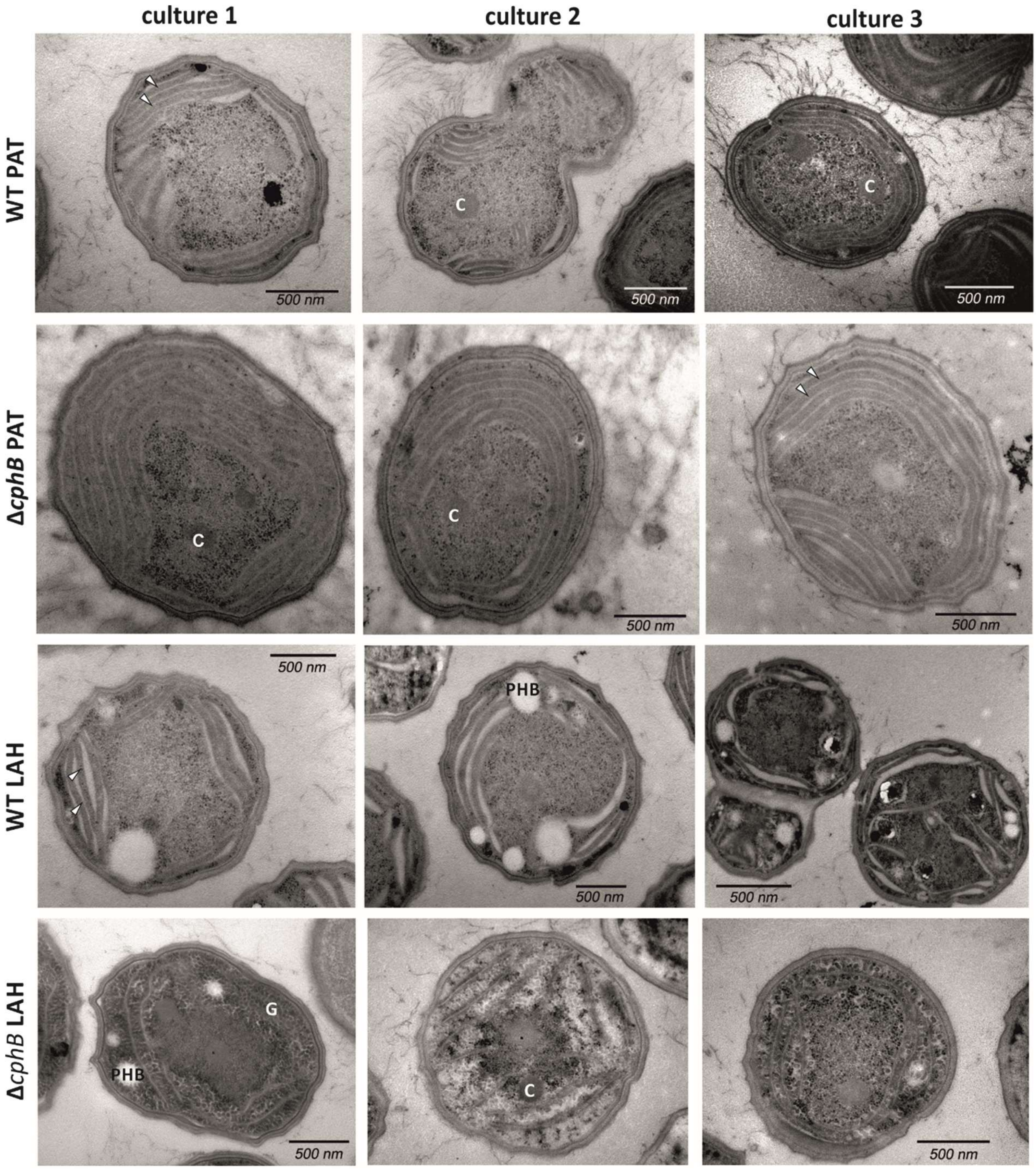

Figure S3 Abnormal accumulation of carbon-storage in cells lacking cyanophycinase (Δ*cphB*) compared to the wild type (WT) control. Transmission electron micrographs were prepared of WT and Δ*cphB* cells that were cultivated in biologically independent, photoautotrophic (PAT) or Light Activated Heterotrophic (LAH) cultures (culture 1-3). For details see Figure 6 in the main text.

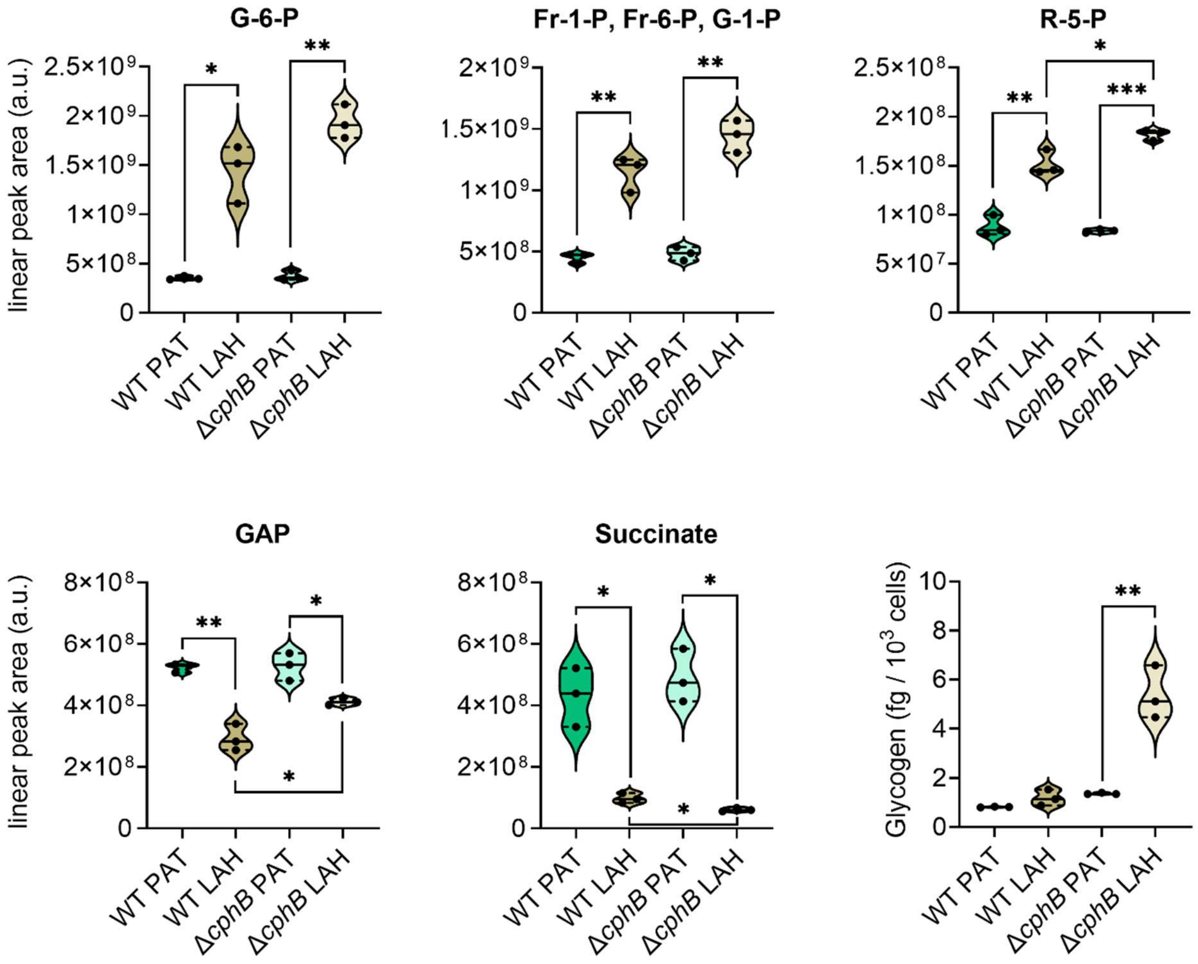

Figure S4 Accumulation of central carbon metabolites in WT and Δ*cphB* during photoautotrophic (PAT) or Light Activated Heterotrophic (LAH) growth. The amounts of the various carbon-phosphates and succinate were determined by LC-HRMS, while glycogen content of the cells was measured according to (55) and (56). Equal amounts of cells were harvested for each sample. The significant differences are indicated. *, $p < 0.05$; **, $p < 0.01$; ***, $p < 0.001$. G-6-P, glucose-6-phosphate; Fr-1-P, Fr-6-P, G-1-P, overlapping peaks of fructose-1-phosphate, fructose-6-phosphate, glucose-1-phosphate; R-5-P, ribose-5-phosphate; GAP, glyceraldehyde-3-phosphate.

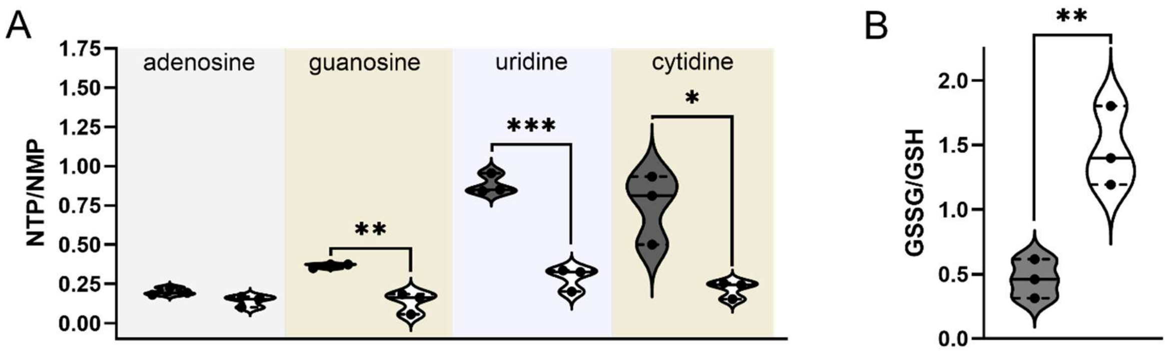

Figure S5 The Δ*cphB* strain synthetize relatively less nucleoside triphosphates and is under higher oxidative stress during the acclimation to Light Activated Heterotrophic growth (LAH). *A*, The amounts of various NTP relative NMP in the WT (grey symbol) and Δ*cphB* (white symbol) cells grown under LAH conditions. *B*, Ratios of the oxidized (GSSG) and reduced (GSH) forms of glutathione in the WT and Δ*cphB* strains in LAH. In panels *A*, and *B*, the amounts of metabolites were determined by LC-HRMS on same amount of cells collected from three independent experiments. The significant differences are indicated; *, $p < 0.05$; **, $p < 0.01$; ***, $p < 0.001$.

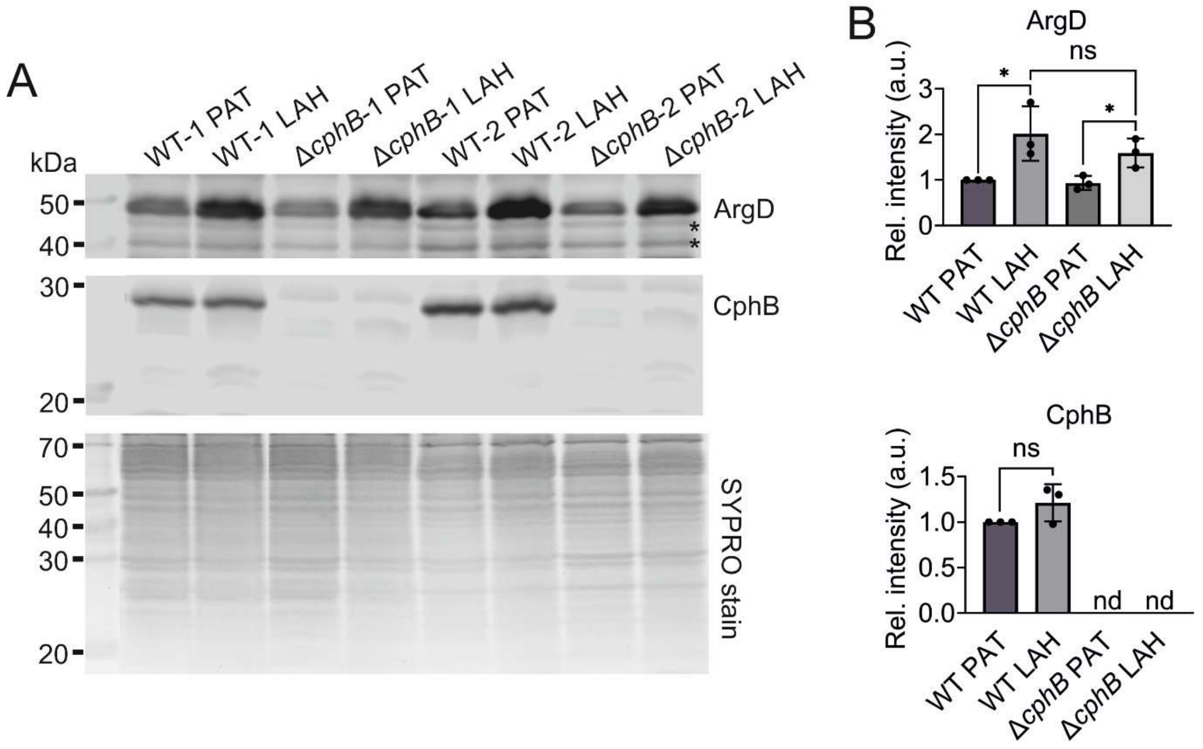

Figure S6 in *vivo* accumulation of the ArgD and CphB enzymes. *A*, The relative levels of ArgD and CphB under photoautotrophic (PAT) or Light Activated Heterotrophic (LAH) in WT and Δ*cphB* were determined by protein immunoblot. Whole cell lysates prepared from biologically independent cultures (designated as 1 and 2) were separated on SDS-PAGE, blotted onto a PVDF membrane, and the ArgD and CphB proteins were detected using specific antibodies. The SYPRO-stained gel is shown for loading control. *, unspecific cross-reaction. *B*, The relative band intensities from the immunoblots presented on panel *A*, and Figure 8A were estimated by ImageJ (53) and are indicated by solid circles. The columns and error bars represent the average intensities and their standard deviations, respectively. The band intensities were normalized to their corresponding loading control, and the band intensity from the PAT-grown control, WT cultures were taken as one. The significance of the differences was evaluated using 2-tailed Student's *t*-test. *, $p < 0.05$; ns, not significant; nd, not detected.

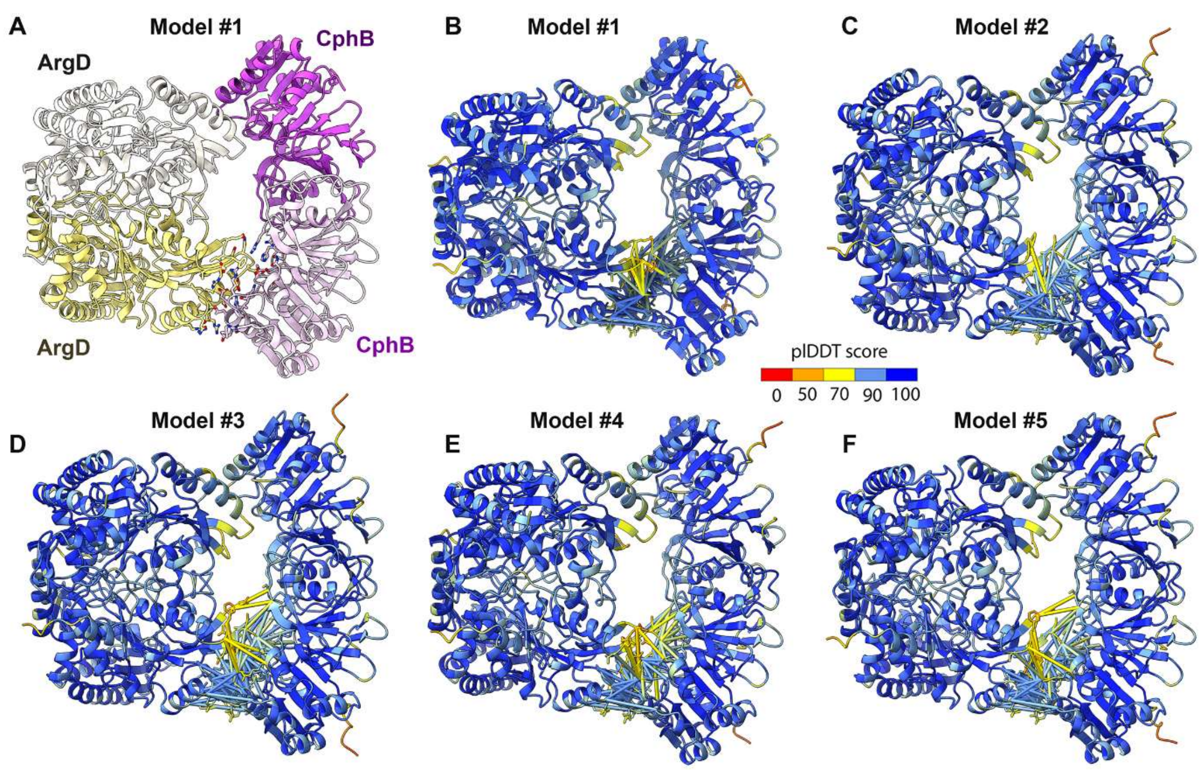

Figure S7 Structural models of the $ArgD_2$-$CphB_2$ complex predicted by AlphaFold 3. Five models are shown, numbered from the highest predicted local distance difference test score (plDDT) (model #1) to the lowest score (model #5). The plDDT score indicates confidence that a structure is predicted accurately *A*, Representation of the individual polypeptides in the structural model #1. *B*, The same model coloured according to the plDDT score, the figure includes also score for contacts (up to 5Å) between CphB and ArgD. *C-F*, The same representation of models #2-#5 as on panel *B*. Images were prepared using ChimeraX (36).

A

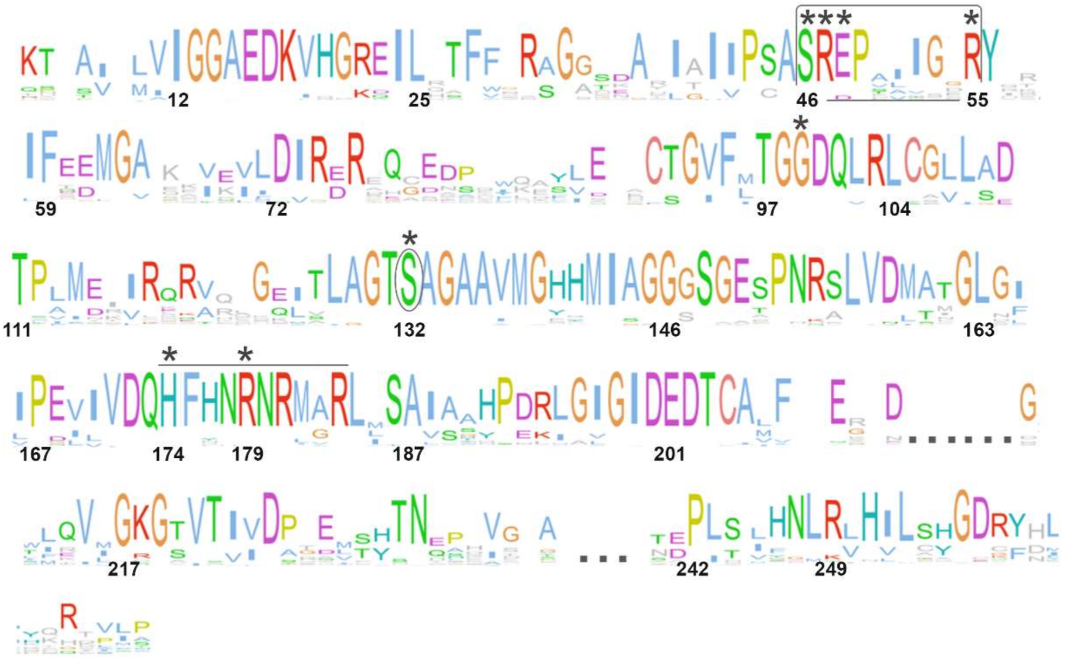

B

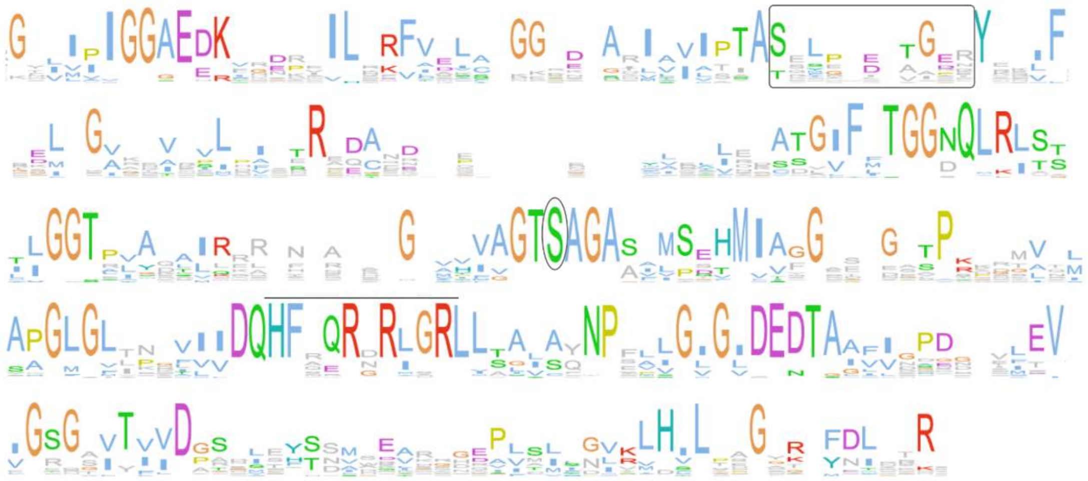

Figure S8 Sequence logos of CphB enzymes. 1000 sequences of *A*, cyanobacterial and *B*, bacterial CphB proteins were collected by BLAST search and aligned in MAFFT (localpair algorithm (111). The resulting alignments were used to construct sequence logos in Jalview 2.11 (112). Residues that, according to AlphaFold 3 prediction, form hydrogen bonds between *Synechocystis* CphB and ArgD (see **Fig. 9C** in main text) are marked by asterisks. The protein segment predicted to be critical for the binding of ArgD is shown in a box. The key serine residue (catalytic nucleophile) of CphB is circled and the substrate-binding motif (34) is marked by horizontal line. Numbering of residues in (A) corresponds to *Synechocystis* CphB.

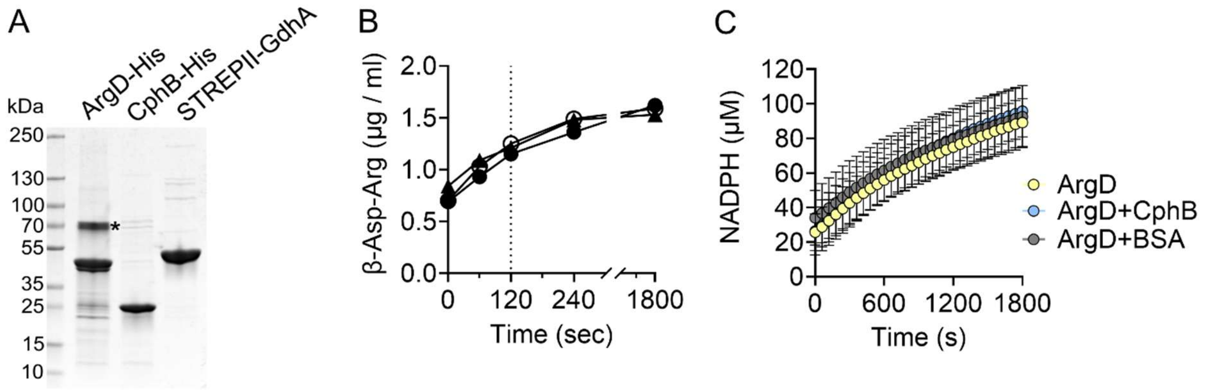

Figure S9 Integrities of the recombinant ArgD and CphB enzymes. *A*, C-terminal His6-tagged ArgD, CphB (ArgD-His, CphB-His), and StrepII-tagged NADP-specific glutamate dehydrogenase (*slr0710*, STREPII-GdhA) of *Synechocystis* were over-expressed and purified form *Escherichia coli*. *, dimeric form of ArgD. *B*, The kinetics of cyanophycin degradation by CphB. The assay started with the addition of 130 µg / ml cyanophycin and the amount of β-Asp-Arg dipeptide generated was measured by UHPLC-MS at the indicated time points. The various symbols represent the independent repetitions of the assay. *B*, NADPH formation in an ArgD and GdhA-coupled enzymatic reaction (37) in the absence or presence of CphB or BSA. The recorded NADPH fluorescence was converted to concentration using NADPH standard curve.

REFERENCES

31. Gonzalez-Esquer, C. R., Smarda, J., Rippka, R., Axen, S. D., Guglielmi, G., Gugger, M., and Kerfeld, C. A. (2016) Cyanobacterial ultrastructure in light of genomic sequence data. *Photosynth Res* **129**, 147-157
34. Sharon, I., Grogg, M., Hilvert, D., and Schmeing, T. M. (2022) The structure of cyanophycinase in complex with a cyanophycin degradation intermediate. *Biochimica et Biophysica Acta (BBA) - General Subjects* **1866**, 130217
36. Meng, E. C., Goddard, T. D., Pettersen, E. F., Couch, G. S., Pearson, Z. J., Morris, J. H., and Ferrin, T. E. (2023) UCSF ChimeraX: Tools for structure building and analysis. *Protein Sci* **32**, e4792
37. Rajaram, V., Ratna Prasuna, P., Savithri, H. S., and Murthy, M. R. N. (2008) Structure of biosynthetic N-acetylornithine aminotransferase from *Salmonella typhimurium*: Studies on substrate specificity and inhibitor binding. *Proteins: Structure, Function, and Bioinformatics* **70**, 429-441
53. Schneider, C. A., Rasband, W. S., and Eliceiri, K. W. (2012) NIH Image to ImageJ: 25 years of image analysis. *Nat Meth* **9**, 671-675
55. Gründel, M., Scheunemann, R., Lockau, W., and Zilliges, Y. (2012) Impaired glycogen synthesis causes metabolic overflow reactions and affects stress responses in the cyanobacterium Synechocystis sp. PCC 6803. *Microbiology (Read)* **158**, 3032-3043
56. Klotz, A., and Forchhammer, K. (2017) Glycogen, a major player for bacterial survival and awakening from dormancy. *Future Microbiology* **12**, 101-104
66. Miyashita, H., Ikemoto, H., Kurano, N., Adachi, K., Chihara, M., and Miyachi, S. (1996) Chlorophyll *d* as a major pigment. *Nature* **383**, 402-402
67. Miyashita, H., Ikemoto, H., Kurano, N., Miyachi, S., and Chihara, M. (2003) *Acaryochloris marina* gen. et. sp. nov. (cyanobacteria), an oxygenic photosynthetic prokaryote containing Chl *d* as a major pigment *J Phycol* **39**, 1247-1253
68. Stanier, R. Y., Kunisawa, R., Mandel, M., and Cohen-Bazire, G. (1971) Purification and properties of unicellular blue-green algae (order *Chroococcales*). *Bacteriol Rev* **35**, 171-205
69. Rippka, R., Deruelles, J., Waterbury, J. B., Herdman, M., and Stanier, R. Y. (1979) Generic assignments, strain histories and properties of pure cultures of cyanobacteria. *Microbiology* **111**, 1-61
70. Moro I, R. N., La Rocca N, Di Bella M, Andreoli C. (2007) *Cyanobacterium aponinum*, a new Cyanoprokaryote from the microbial mat of Euganean thermal springs (Padua, Italy). *Algological Studies* **123**, 1-15
71. Lin, J.-Y., and Ng, I. S. (2023) Thermal cultivation of halophilic *Cyanobacterium aponinum* for *c*-phycocyanin production and simultaneously reducing carbon emission using wastewater. *Chem Eng J* **461**, 141968
72. Rippka, R., and Cohen-Bazire, G. (1983) The cyanobacteriales: A legitimate order based on the type strain *Cyanobacterium stanieri*? *Annales de l'Institut Pasteur / Microbiologie* **134**, 21-36
73. Gerloff, G. C., Fitzgerald, G.P., and Skoog, F. (1950) The isolation, purification and nutrient solution requirements of blue-green algae." In: Proceedings of the Symposium on the Culturing of Algae. *Dayton, Ohio, USA: Charles F. Kettering Foundation*, 44
74. Robertson, B. R., Tezuka, N., and Watanabe, M. M. (2001) Phylogenetic analyses of Synechococcus strains (Cyanobacteria) using sequences of 16S rDNA and part of the phycocyanin operon reveal multiple evolutionary lines and reflect phycobilin content. *Int J Syst Evol Microbiol* **51**, 861-871
75. Laloui, W., Palinska, K. A., Rippka, R., Partensky, F., deMarsac, N. T., Herdman, M., and Iteman, I. (2002) Genotyping of axenic and non-axenic isolates of the genus *Prochlorococcus* and the OMF-'*Synechococcus*' clade by size, sequence analysis or RFLP of the internal transcribed spacer of the ribosomal operon. *Microbiology SGM* **148**, 453-465

76. Ernst, A., Becker, S., Wollenzien, U. I. A., and Postius, C. (2003) Ecosystem-dependent adaptive radiations of picocyanobacteria inferred from 16S rRNA and ITS-1 sequence analysis. *Microbiology* **149**, 217-228
77. Bandyopadhyay, A., Elvitigala, T., Welsh, E., Stockel, J., Liberton, M., Min, H. T., Sherman, L. A., and Pakrasi, H. B. (2011) Novel metabolic attributes of the genus *Cyanothece*, comprising a group of unicellular nitrogen-fixing cyanobacteria. *Mbio* **2**
78. Welsh, E. A., Liberton, M., Stoeckel, J., Loh, T., Elvitigala, T., Wang, C., Wollam, A., Fulton, R. S., Clifton, S. W., Jacobs, J. M., Aurora, R., Ghosh, B. K., Sherman, L. A., Smith, R. D., Wilson, R. K., and Pakrasi, H. B. (2008) The genome of *Cyanothece* 51142, a unicellular diazotrophic cyanobacterium important in the marine nitrogen cycle. *Proc Natl Acad Sci USA* **105**, 15094-15099
79. Reddy, K. J., Haskell, J. B., Sherman, D. M., and Sherman, L. A. (1993) Unicellular, aerobic nitrogen-fixing cyanobacteria of the genus *cyanothece*. *J Bacteriol* **175**, 1284-1292
80. Garlick, S., Oren, A., and Padan, E. (1977) Occurrence of facultative anoxygenic photosynthesis among filamentous and unicellular cyanobacteria. *J Bacteriol* **129**, 623-629
81. Otsuka, S., Suda, S., Li, R., Watanabe, M., Oyaizu, H., Matsumoto, S., Watanabe, and M., M. (1999) Characterization of morphospecies and strains of the genus *Microcystis* (Cyanobacteria) for a reconsideration of species classification. *Phycol Res* **47**, 189-197
82. Kratz, W. A., and Myers, J. (1955) Nutrition and growth of several blue-green algae. *Am J Bot* **42**, 282-287
83. Grigorieva GA, S. S. (1979) Application of the genetic transformation method for taxonomic analysis of unicellular blue-green algae. in *Proceedings of the 2nd International Symposium on Photosynthetic Prokaryotes* (Codd GA, S. W. ed.
84. Shestakov, S. V. a. R., J. (1987) Gene transfer and host-vector systems of cyanobacteria. *Oxf Surv Plant Mol Cell Biol* **4**, 137-166
85. Li, Y., Rao, N. N., Yang, Y., Zhang, Y., and Gu, Y. N. (2015) Gene annotation and functional analysis of a newly sequenced *Synechococcus* strain. *Genet Mol Res* **14**, 12416-12426
86. Waterbury, J. B., and Stanier, R. Y. (1978) Patterns of growth and development in pleurocapsalean cyanobacteria. *Microbiol Rev* **42**, 2-44
87. Garcia-Pichel, F., Prufert-Bebout, L., and Muyzer, G. (1996) Phenotypic and phylogenetic analyses show *Microcoleus chthonoplastes* to be a cosmopolitan cyanobacterium. *Appl Environ Microbiol* **62**, 3284-3291
88. Siegesmund, M. A., Johansen, J. R., Karsten, U., and Friedl, T. (2008) *Coleofasciculus* gen. nov. (cyanobacteria): morphological and molecular criteria for revision of the genus *Microcoleus gomont* (1) *J Phycol* **44**, 1572-1585
89. Rippka, R., and Herdmann, H. (1992) Pasteur culture collection of cyanobacterial strains in axenic culture. Cyanobacteria catalogue & taxonomic handbook. Vol. I. Catalogue of strains 1992/1993. Paris: Institut Pasteur.
90. Shih, P. M., Wu, D., Latifi, A., Axen, S. D., Fewer, D. P., Talla, E., Calteau, A., Cai, F., Tandeau de Marsac, N., Rippka, R., Herdman, M., Sivonen, K., Coursin, T., Laurent, T., Goodwin, L., Nolan, M., Davenport, K. W., Han, C. S., Rubin, E. M., Eisen, J. A., Woyke, T., Gugger, M., and Kerfeld, C. A. (2013) Improving the coverage of the cyanobacterial phylum using diversity-driven genome sequencing. *Proc Natl Acad Sci U S A* **110**, 1053-1058
91. Baalen, C. v. (1962) Studies on marine blue-green algae. *Bot Mar* **4**, 129-139
92. Perkerson Iii, R. B., Johansen, J. R., Kovácik, L., Brand, J., Kaštovský, J., and Casamatta, D. A. (2011) A unique *pseudanabaenalean* (cyanobacteria) gens *nodosilinea* gen. nov. based on morphological and molecular data. *J Phycol* **47**, 1397-1412
93. Lachance, M.-A. (1981) Genetic relatedness of heterocystous cyanobacteria by deoxyribonucleic acid-deoxyribonucleic acid reassociation. *Int J Syst Evol Microbiol* **31**, 139-147

94. Kenyon, C. N., Rippka, R., and Stanier, R. Y. (1972) Fatty acid composition and physiological properties of some filamentous blue-green algae. *Archiv für Mikrobiologie* **83**, 216-236
95. Ekman, M., Picossi, S., Campbell, E. L., Meeks, J. C., and Flores, E. (2013) A Nostoc punctiforme sugar transporter necessary to establish a cyanobacterium-plant symbiosis *Plant Physiol* **161**, 1984-1992
96. Gagunashvili, A. N., and Andrésson, Ó. S. (2018) Distinctive characters of Nostoc genomes in cyanolichens. *BMC Genomics* **19**, 434
97. Adolph, K. W., and Haselkorn, R. (1971) Isolation and characterization of a virus infecting the blue-green alga *Nostoc muscorum*. *Virology* **46**, 200-208
98. Stebegg, R., Wurzinger, B., Mikulic, M., and Schmetterer, G. (2012) Chemoheterotrophic growth of the cyanobacterium *Anabaena* sp. strain PCC 7120 dependent on a functional cytochrome c oxidase. *J Bacteriol* **194**, 4601-4607
99. Kettler, G. C., Martiny, A. C., Huang, K., Zucker, J., Coleman, M. L., Rodrigue, S., Chen, F., Lapidus, A., Ferriera, S., Johnson, J., Steglich, C., Church, G. M., Richardson, P., and Chisholm, S. W. (2007) Patterns and implications of gene gain and loss in the evolution of *Prochlorococcus*. *PLoS genetics* **3**, e231
100. Biller, S. J., Berube, P. M., Berta-Thompson, J. W., Kelly, L., Roggensack, S. E., Awad, L., Roache-Johnson, K. H., Ding, H., Giovannoni, S. J., Rocap, G., Moore, L. R., and Chisholm, S. W. (2014) Genomes of diverse isolates of the marine cyanobacterium *Prochlorococcus*. *Scientific Data* **1**, 140034
101. Walter, J. M., Coutinho, F. H., Dutilh, B. E., Swings, J., Thompson, F. L., and Thompson, C. C. (2017) Ecogenomics and taxonomy of cyanobacteria phylum. *Frontiers in Microbiology* **8**
102. Scanlan, D. J., Mann, N. H., and Carr, N. G. (1993) The response of the picoplanktonic marine cyanobacterium *Synechococcus species* WH7803 to phosphate starvation involves a protein homologous to the periplasmic phosphate-binding protein of *Escherichia coli*. *Mol Microbiol* **10**, 181-191
103. Palenik, B. (2012) Recent functional genomics studies in marine *Synechococcus*. in *Functional genomics and evolution of photosynthetic systems* (Burnap, R., and Vermaas, W. eds.), Springer Netherlands, Dordrecht. pp 103-118
104. Toledo, G., Palenik, B., and Brahamsha, B. (1999) Swimming marine *Synechococcus* strains with widely different photosynthetic pigment ratios form a monophyletic group. *Appl Environ Microbiol* **65**, 5247-5251
105. Coutinho, F. H., Dutilh, B. E., Thompson, C. C., and Thompson, F. L. (2016) Proposal of fifteen new species of *Parasynechococcus* based on genomic, physiological and ecological features. *Arch Microbiol* **198**, 973-986
106. Walworth, N., Pfreundt, U., Nelson, W. C., Mincer, T., Heidelberg, J. F., Fu, F., Waterbury, J. B., Glavina del Rio, T., Goodwin, L., Kyrpides, N. C., Land, M. L., Woyke, T., Hutchins, D. A., Hess, W. R., and Webb, E. A. (2015) Trichodesmium genome maintains abundant, widespread noncoding DNA in situ, despite oligotrophic lifestyle. *Proc Natl Acad Sci U S A* **112**, 4251-4256
107. Prufert-Bebout, L., Paerl, H. W., and Lassen, C. (1993) Growth, nitrogen fixation, and spectral attenuation in cultivated *Trichodesmium* species. *Appl Environ Microbiol* **59**, 1367-1375
108. Nakamura, Y., Kaneko, T., Sato, S., Ikeuchi, M., Katoh, H., Sasamoto, S., Watanabe, A., Iriguchi, M., Kawashima, K., Kimura, T., Kishida, Y., Kiyokawa, C., Kohara, M., Matsumoto, M., Matsuno, A., Nakazaki, N., Shimpo, S., Sugimoto, M., Takeuchi, C., Yamada, M., and Tabata, S. (2002) Complete genome structure of the thermophilic cyanobacterium *Thermosynechococcus elongatus* BP-1. *DNA Research* **9**, 123-130
109. Zilliges, Y., and Dau, H. (2016) Unexpected capacity for organic carbon assimilation by *Thermosynechococcus elongatus*, a crucial photosynthetic model organism. *FEBS Lett* **590**, 962-970
110. Adhikary, S. P., and Pattnaik, H. (1979) Growth response of *Westiellopsis prolifica janet* to organic substrates in light and dark. *Hydrobiologia* **67**, 241-247

111. Katoh, K., and Standley, D. M. (2013) MAFFT multiple sequence alignment software version 7: improvements in performance and usability. *Mol Biol Evol* **30**, 772-780
112. Waterhouse, A. M., Procter, J. B., Martin, D. M. A., Clamp, M., and Barton, G. J. (2009) Jalview Version 2—a multiple sequence alignment editor and analysis workbench. *Bioinformatics* **25**, 1189-1191
